# Unexpected Suppression of Double-Proton Tunneling Induced by Quantum Barriers from Zero-Point Energy

**DOI:** 10.1021/acs.joc.5c00827

**Published:** 2025-07-17

**Authors:** Edyta M. Greer, Florence Uritsky, Benjamin Herrera, Frankie Benavides, Alexander Greer, Charles Doubleday

**Affiliations:** † Department of Natural Sciences, Baruch College of the City University of New York, New York, New York 10010, United States; ‡ Department of Chemistry, Brooklyn College, Brooklyn, New York 11210, United States; ∥ Ph.D. Program in Chemistry, The Graduate Center of the City University of New York, New York, New York 10016, United States; § Department of Chemistry, 5798 Columbia University, New York, New York 10027, United States

## Abstract

To assess tunneling, we studied the guanine–cytosine (GC) base pair tautomerization in the gas phase. We applied multidimensional semiclassical reaction path methodology with microcanonically optimized multidimensional tunneling (μOMT) using POLYRATE. The minimum energy path (MEP) has a single saddle point for the double proton transfer. Addition of vibrational zero-point energy (ZPE) to the MEP gives the vibrationally adiabatic ground state curve, *V*_a_^G^, which is the barrier through which tunneling occurs. Unexpectedly, *V*_a_^G^ has not one but two well-separated barriers in the transition state region. The first is near the saddle point. The second barrier is entirely due to a large amount of ZPE associated with local reaction path curvature. We refer to it as a quantum barrier. Its height and width reduce the tunneling transmission coefficient. In other words, GC tautomerization has two competing quantum effects, tunneling and ZPE, that have opposite effects on the reaction rate. The transmission coefficient κ is 1.57, and tunneling constitutes 36% of the rate constant at 298 K. Our computed kinetic isotope effects (KIE) are lower than expected, e.g., KIE = 5.05 at 298 K. In the discussion, we show that the quantum barrier is a consequence of reaction path curvature as the tautomer begins to form.

## Graphical abstract

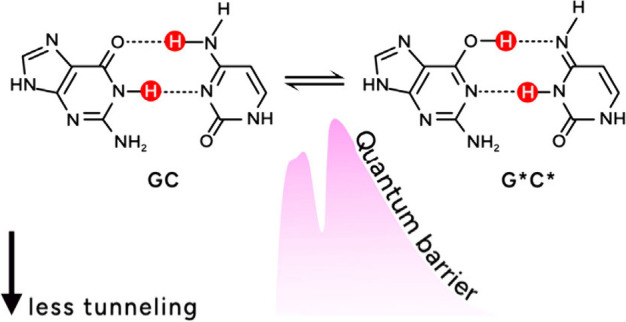

## Introduction

Our goal is to compute the contribution of quantum tunneling to the rate of tautomeric isomerization of the guanine–cytosine (GC) DNA base pair (Figure 1).^1,2^ To achieve this, we use POLYRATE^3^ to compute the height and shape of the reaction path and the tunneling transmission coefficient, κ.^4^

**Figure 1:**
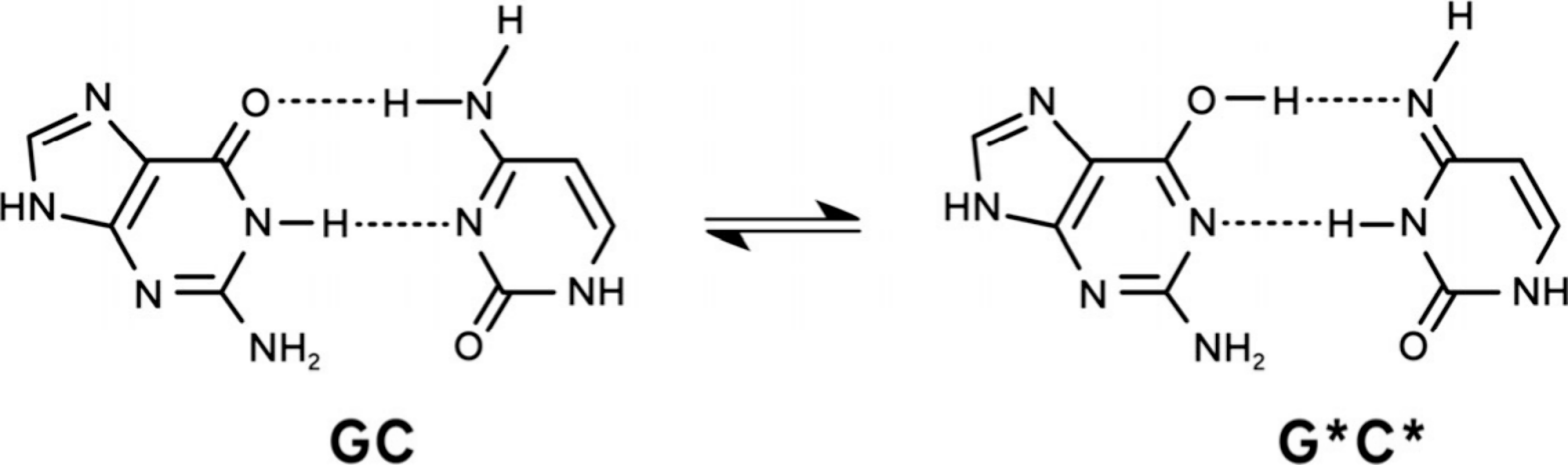
Isolated GC and double-proton transfer in the tautomeric G*C* form.

The reaction path has an unexpected feature that merits a discussion here. The path is determined by following the minimum energy path (MEP) of the electronic energy, which connects the transition state (TS) to the product in the forward direction and to the reactant in the reverse direction. At each point along this path, a quantum correction is applied by adding its vibrational zero-point energy (ZPE). The ZPE-corrected MEP is referred to as *V*_a_^G^ (vibrationally adiabatic ground state), which represents the barrier through which tunneling occurs. Typically, *V*_a_^G^ closely follows the MEP at a nearly constant energy above the MEP.^4^ However, in this case, we observe that *V*_a_^G^ has two maxima: one near the saddle point and another higher maximum on the product side of the saddle point (Figure 2). We refer to this second barrier as a “quantum barrier”, as it arises from a local increase in ZPE, which we discuss below.

**Figure 2:**
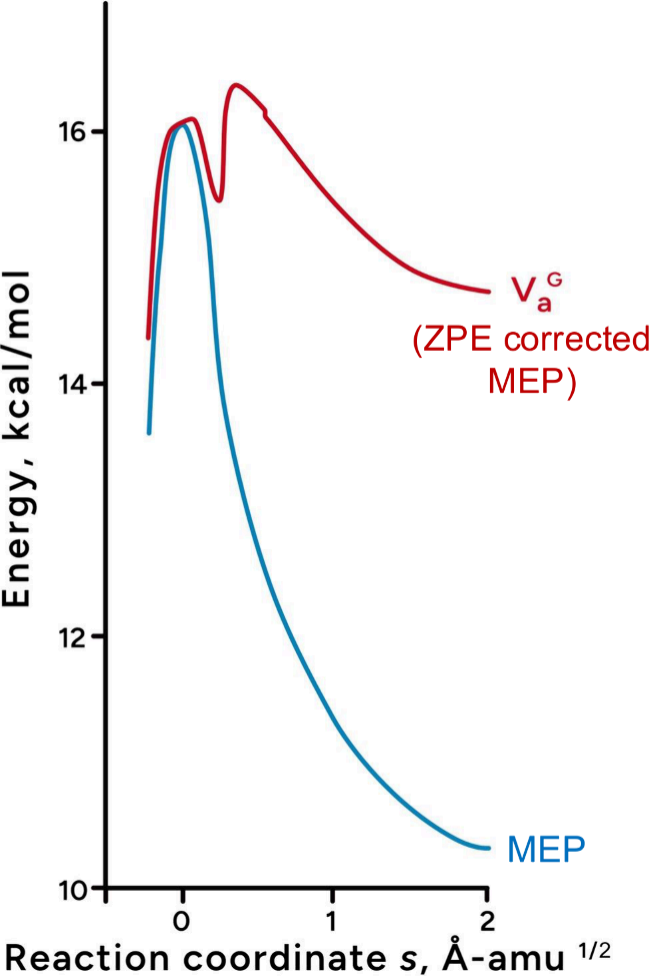
MEP vs *V*_a_^G^ for GC → G*C*. For ease of comparison, *V*_a_^G^ values are normalized to MEP at the saddle point *s* = 0.

In our recent study of tunneling in the biosynthesis of tetrahydrocannabinol (THC), one of the key steps involved tautomerization with double proton transfer, as illustrated in Figure 3A.^5^ To explore tunneling effects, vibrational zero-point energy (ZPE) corrections were calculated at regular intervals along the minimum energy path (MEP), which yielded the vibrationally adiabatic ground-state potential, *V*_a_^G^,^4^ as shown qualitatively in Figure 3B. Contrary to our expectation of a single maximum, we observed two maxima: one at the dynamical bottleneck near the saddle point and another smaller barrier and a *hidden* intermediate^6^ state between them. This second barrier, illustrated in Figure 3B, is the result of variations in ZPE during the double-proton transfer, creating a “quantum barrier”. This quantum barrier significantly widened the overall energy barrier and suppressed tunneling.

**Figure 3:**
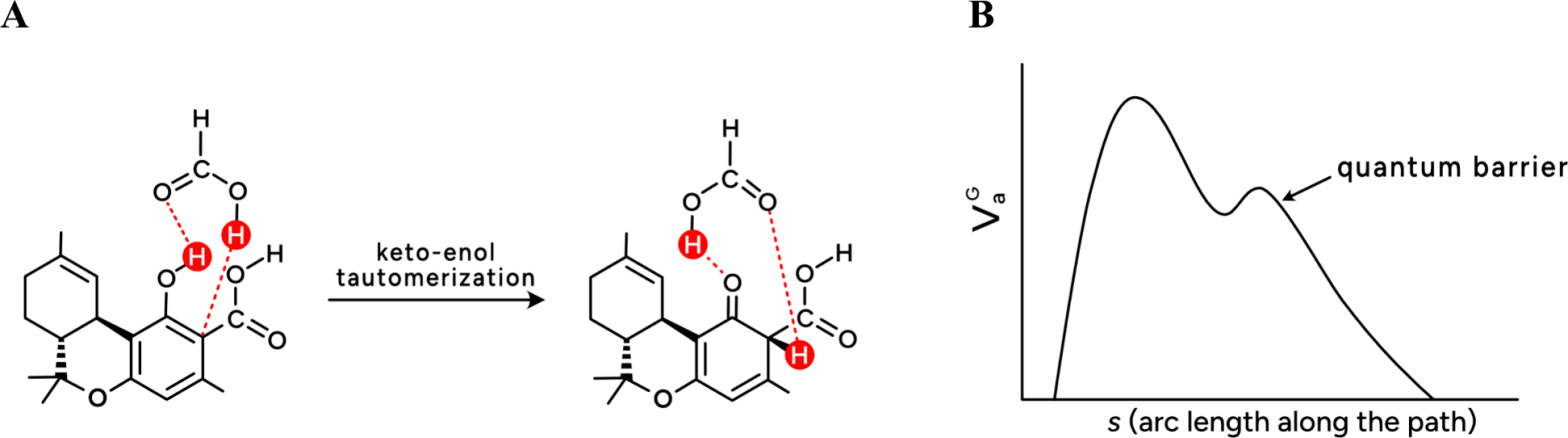
(A) Effect of one formic acid molecule on double proton transfer in a model THC compound. Here, THC has an *n*-pentyl chain replaced by methyl. (B) Qualitative *V*_a_^G^ curve for the reaction is shown in Figure 3A.

This observation suggests that the double-barrier potential, which includes a saddle point on the potential energy surface and a nearby quantum barrier, increases the width of the full barrier and decreases the extent of tunneling. This mechanism could be particularly significant in tautomeric processes that involve a double proton transfer. To explore this, we focused on the tautomeric equilibrium in the DNA base pairs. For this investigation, we specifically examined the isolated GC base pair and its tautomer, G*C*,^1,2^ as shown in Figure 1. Our findings reveal that double proton transfer in GC involves competing quantum effects, namely, tunneling and vibrational zero-point energy (ZPE).

## Computational Methodology

Electronic structure calculations were carried out with Gaussian 16 ^7^ using the dispersion corrected ωB97XD^8^ functional and 6-311+G­(d,p) basis set. Early quantum chemical studies of biomolecules reported charge distributions, resonance energies, and stabilizations arising from the van der Waals–London interactions.^9^ In this project, we use ωB97XD/6-311+G­(d,p) since it has been successful in understanding details of DNA base pairing.^10^ Transition states were located variationally with CVT/CAG,^11–13^ and the minimum energy path (MEP) on the Born–Oppenheimer potential energy surface (PES) was computed using POLYRATE^3^ with the GAUSSRATE^14^ interface to Gaussian 16.^7^ POLYRATE handles systems with multiple barriers along the reaction path.^15–19^ The minimum energy ωB97XD/6-311+G­(d,p) path is modified by adding a zero-point energy at every point of the MEP to give the barrier through which the tunneling occurs, which is the vibrationally adiabatic ground-state curve, *V*_a_^G^.

We elected to carry out calculations in the gas phase.^20^ Solvent calculations of isolated GC^21,22^ or GC embedded in a DNA fragment^22,23^ can change the mechanism from concerted to stepwise. We did not address the issue of solvation nor did we model the effects of the polymerase or helicase. Key insights into these factors have been previously found.^24–26^ Instead, to limit the number of variables and establish a control for future studies, we chose to focus solely on the gas-phase effects of the GC ⇄ G*C* system.

Previous computational work has been performed to investigate the role of quantum effects in double-proton transfers in GC or related systems with other computational methods, including: (1) path integral molecular dynamics showing the tautomeric G*C* form as a source of DNA mutations to be unlikely.^23,27^ Also, acceleration of tautomerization^23,27^ is likely, but the mechanism is tuned by the biological environment.^23^ (2) Machine learning-nudged elastic band (ML-NEB) algorithm results showed tunneling correction = 1.222 × 10^2^, a sizable G*C* ⇄ GC barrier, and G*C* lifetime τ_r_ = 1.63 × 10^–11^ s; thus, it is thought to be long enough for development of point mutations.^28^ (3) The open quantum system (OQS) method gave tunneling coefficient κ = ∼10^5^ and kinetic isotope effect KIE = 30 for GC → G*C*, characteristic of tunneling.^29^

To explore tunneling in the GC ⇄ G*C* equilibrium, we employed variational transition state theory (VTST)^30^ combined with microcanonically optimized multidimensional tunneling (μOMT) corrections^4,30^ to the rate constant, as implemented in POLYRATE. This approach applies VTST with multidimensional curvature and tunneling, which has not previously been used to study the GC ⇄ G*C* system. This methodology has been successfully validated in various organic, bioorganic, and enzymatic systems through comparisons with experimental results.^5,31–42^

The following POLYRATE options were used: MEP was calculated by the Page-McIver algorithm [RPM = pagem][Bibr ref43] with a step size of 0.002 Å-amu^1/2^ in isoinertial coordinates [coord = curv3]. The Hessian calculations were carried out every 10 steps. The reaction path degeneracy was assumed to be 1 since the path is unique and contains no bifurcation. A scale factor of 1 was used to scale the harmonic frequencies obtained with ωB97XD/6-311+G­(d,p). The rate constant is given by

$$k=\kappa \frac{k_{B}T}{h}\;\exp\left(-\frac{\Delta G^{‡}}{RT}\right)$$

where κ is the transmission coefficient computed by μOMT, *k*_B_ is Boltzmann’s constant, *R* is the gas constant, *T* is temperature, and Δ*G* ^‡^ is the free energy of activation. Rate constants were computed over the 200–450 K temperature range with CVT/CAG.^11–13^ The transmission coefficient for the tunneling correction was calculated by μOMT.^44^ The μOMT correction selects the largest value between the small-curvature tunneling (SCT)^45–47^ and the large-curvature tunneling (LCT),^44,46–50^ version 4.^48^ The interpolated large-curvature tunneling algorithm in two dimensions [ILCT2D] was utilized to create a 2D grid [LCTGRID].^19^ This LCTGRID was used to calculate the LCT transmission probabilities used in both the LCT and μOMT approximations. Default options were used where the 2D grid contained 9 grid points for the energy coordinate and 11 grid points for the tunneling coordinate.^51^ The available vibrational excited states were all included for tunneling contributions.^52^ In POLYRATE, the transmission coefficient is calculated for the ground state of the reactant in the exoergic direction. Then, the transmission coefficient for the endoergic direction is obtained by a detailed balance, which requires κ for a thermal reaction to be the same in both directions.

Finally, we calculated a lack of contributions of tunneling in tautomerization of adenine–thymine (AT) due to high instability of the tautomeric form A*T* (see Figure S2, SI).^53^ This result is in contrast to GC, in which GC tautomerization produces a barrier.

## Results and Discussion

Here, we present our computed results to advance the state of the art tunneling assessments on the free-energy profile, geometries of GC and G*C*, transmission coefficients, kinetic evaluation, and KIE and Arrhenius plots, followed by a summary section.

### Free-Energy Profile

Figure 4 shows the classical free energy profile for the intermolecular double proton transfer in GC obtained with ωB97XD/6-311+G­(d,p) at 298 K in the gas phase. In Figure 4, a barrier is found where the TS and product G*C* are similar, as would be expected of a late TS (Hammond’s postulate).^54^ Here, the free-energy difference between GC and G*C* is 10.4 kcal/mol, wherein the forward and reversed barriers show the tautomerization of the GC to G*C* and are 12.9 and 2.5 kcal/mol, respectively. Notice that there is a double proton transfer (not triple proton transfer) arising in going from GC to G*C*. This is an asynchronous double proton transfer, where the proton in N1–H···N3 transfers first and is followed by the proton in O6···H–N4. This occurs in a single-step process defined by one transition state.

**Figure 4:**
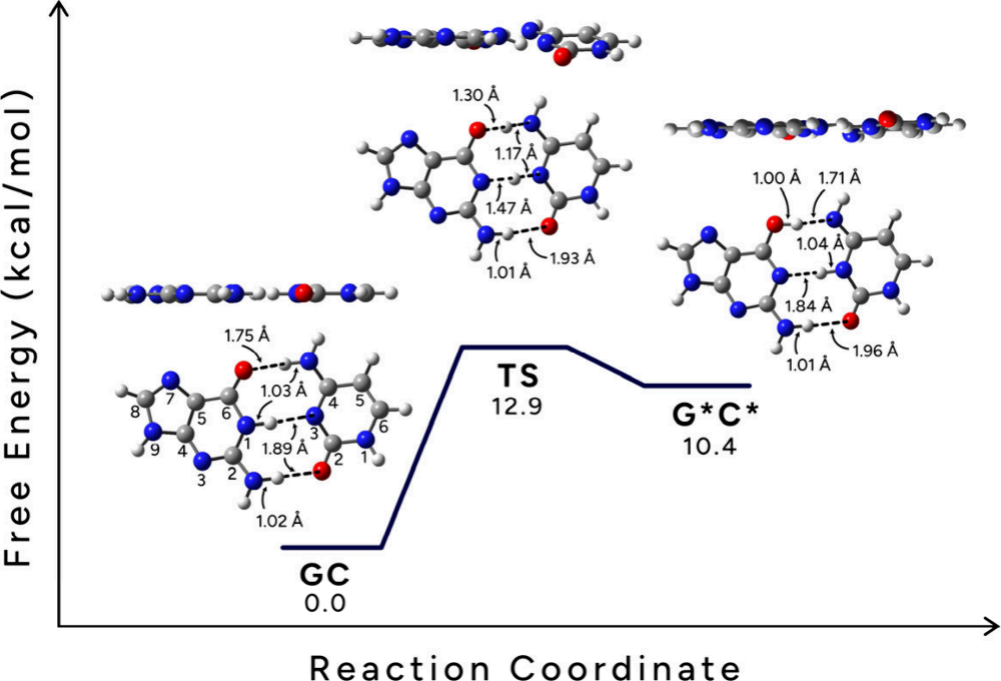
Gas phase ωB97XD/6-311+G­(d,p) free energy surface (PES) for tautomerization of the GC base pair. Side and top view of GC, TS, G*C*, and free energy (G) at 298 K.

### Geometries

The GC pair is planar, but the TS and G*C* pairs are slightly distorted (Figure 4). The distances between heavy atoms of the hydrogen bonds in the GC, TS, and G*C* are in excellent agreement with ωB97XD/6-311+G­(d,p) compared to values obtained with B3LYP/6-311++G­(d,p) suggesting that our theoretical method is working well.^55^

### Transmission Coefficients (κ)

The transmission coefficients were calculated by CVT/CAG and μOMT for GC → G*C* and for double deuterated GC, *dd*GC → *dd*G*C* (Table 1). In Table 1, GC → G*C* tunneling is found to be the greatest at 200–298 K with κ_μ__OMT_ = 2.52 at 200 K and lower at 298 K with κ_μ__OMT_ = 1.57. The calculation reveals that tunneling enhances the CVT/CAG rate of GC → G*C* by 36% at 298 K. We use (κ – 1)/κ as a measure of the fraction of the reaction attributable to tunneling (κ_μ__OMT_ = 1 means no tunneling). The κ_μ__OMT_ values are similar to those of κ_LCT_, but both are higher than those of κ_SCT_ (Table S1, SI). For example, at 200 K, κ_μ__OMT_ is slightly higher than κ_LCT_, by 0.12, but much higher than κ_SCT_, by 0.37, indicating that the process is dominated by a large curvature tunneling. In Table 1, *dd*GC → *dd*G*C* tunneling is found to be the greatest at 200 to 298 K: κ_μ__OMT_ = 4.79 at 200 K and κ_μ__OMT_ = 2.13 at 298 K. Because κ_μ__OMT_ values are identical with κ_SCT_ values and both are higher than κ_LCT_, this indicates a process dominated by a small curvature tunneling (Table S4, SI). Noticeably, the κ_μ__OMT_ values for GC → G*C* are much smaller than those for *dd*GC → *dd*G*C*. This can be explained on the basis of the shape of *V*_a_^G^. In GC → G*C*, tunneling occurs through two barriers, whereas in *dd*GC → *dd*G*C* tunneling mostly occurs through a single barrier (Figures 5 and 7, *vide infra*).

**Table 1:** Calculated CVT/CAG and μOMT Transmission Coefficients, κ, for the Tautomerization GC → G*C* and *dd*GC → *dd*G*C* at Selected Temperatures^a^

	GC → G*C*		*dd*GC → *dd*G*C*	
*T* [K]	κ_CVT/CAG_ ^b^	κ_μ__OMT_	κ_CVT/CAG_ ^b^	κ_μ__OMT_
200	0.42	2.52	0.92	4.79
273	0.52	1.70	0.92	2.44
298	0.54	1.57	0.92	2.13
333	0.57	1.45	0.92	1.85
373	0.61	1.35	0.91	1.65
400	0.62	1.30	0.91	1.55
450	0.65	1.23	0.91	1.42

a Additional temperatures are shown in the Supporting Information.

b κ_CVT/CAG_ = exp­{β­[*V*_a_^G^ (s_*_^CVT^ (*T*)) – *V* ^AG^]}. For instances, when κ_CVT/CAG_ < 1, the top of ZPE corrected MEP (*V* ^AG^) is higher in energy than the maximum of the free energy at temperature *T* at the value of s_*_^CVT^ (*T*).^11^

**Figure 5:**
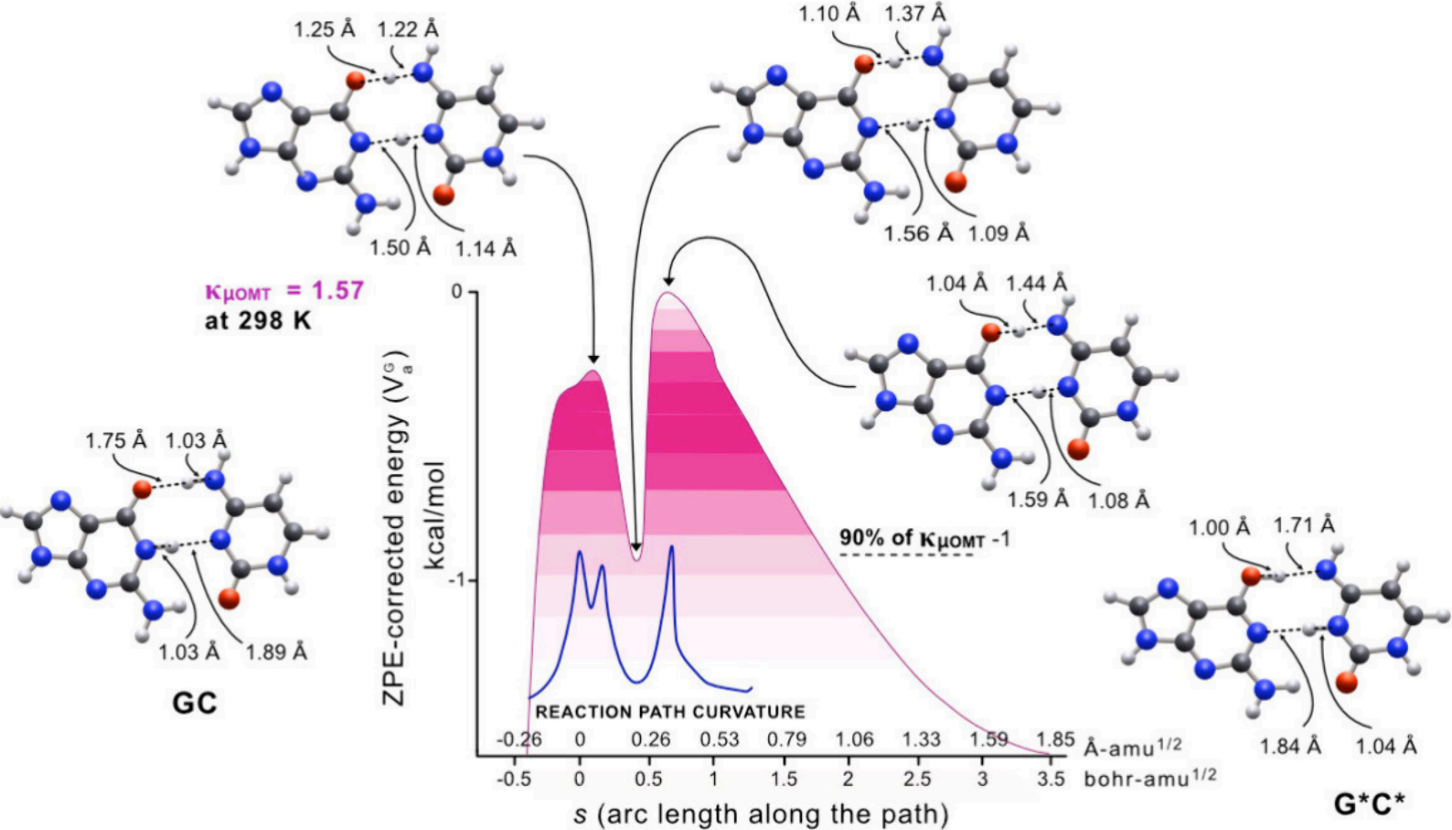
ZPE-corrected MEP (*V*_a_^G^ curve), where 0 of the energy is the highest point on *V*_a_^G^ for GC → G*C*.

Figure 5 shows the degree of tunneling contributions with increasing color intensity in energy slices. Each color intensity increase is proportional to its contribution to the transmission coefficient κ_μ__OMT_. The combined light and dark magenta region is the highest color intensity in which 90% of tunneling occurs within ∼1 kcal/mol of the top of the barrier. Notice that there are two barriers on the *V*_a_^G^ with maxima at *s* = 0.0598 Å-amu^1/2^ and *s* = 0.360 Å-amu^1/2^, respectively, due to the large curvature. This large curvature is shown with the blue curve superimposed on the *V*_a_^G^ curve. The left-hand (lower and narrower) barrier of *V*_a_^G^ is MEP dominated. The right-hand (taller) barrier is due to variations in the ZPE, which we refer to as a quantum barrier. The sum of the widths of both barriers in Figure 5 is three times greater than the MEP (Figure 2), which accounts for the severe suppression of tunneling in Figure 5. Contributions of tunneling decrease with the increase of barrier width.^56,57^ With the κ data in hand, kinetics evaluation is an important next step.

### Kinetic Evaluation

Rate constants for the tautomerization of GC with CVT/CAG as well as those including SCT, LCT, and μOMT are provided in Table S1. In Table S1, due to the large reaction curvature, computations that included μOMT are important, because μOMT selects the larger of SCT and LCT. Tautomerizations at all temperatures are faster when tunneling is included, and the rate constants with tunneling contributions including *k*_*f*,H_^CVT/SCT^, *k*_*f*,H_^CVT/LCT^, and *k*_*f*,H_^CVT/μOMT^ are larger than the rate constants *k*_*f*,H_^CVT/CAG^, computed with CVT/CAG.^11^ Notice that exchanging transferring hydrogens in GC for deuterium substitution in *dd*GC leads to rates *k*_*f*,D_^CVT/SCT^, *k*_*f*,D_^CVT/LCT^, and *k*_*f*,D_^CVT/μOMT^ that are lower for all tunneling corrections but yet are still higher than rate constants with no tunneling corrections. Next, Table 2 shows the computed lifetime (τ) data.

**Table 2:** Forward Rate Constants *k*_*f*_ and Lifetimes τ_*f*_ and Reverse Rate Constants *k*_*r*_ and Lifetimes τ_*r*_ with (μOMT) and without (CAG/CVT) Tunneling for GC to G*C*

*T* [K]	*k*_*f*,H_^CVT/CAG^ [s^–1^]	τ_*f*,H_^CVT/CAG^ [s]	*k*_*f*,H_^CVT/μOMT^ [s^–1^]	τ_*f*,H_^CVT/μOMT^ [s]	*k*_*r*,H_^CVT/CAG^ [s^–1^]	τ_*r*,H_^CVT/CAG^ [s]	*k*_*r*,H_^CVT/μOMT^ [s^–1^]	τ_*r*,H_^CVT/μOMT^ [s]
200	4.91 × 10^–2^	2.03 × 10^1^	1.24 × 10^–1^	8.08	7.13 × 10^9^	1.40 × 10^–10^	1.80 × 10^10^	5.57 × 10^–11^
273	1.60 × 10^2^	6.27 × 10^–3^	2.72 × 10^2^	3.68 × 10^–3^	2.83 × 10^10^	3.53 × 10^–11^	4.82 × 10^10^	2.07 × 10^–11^
298	1.02 × 10^3^	9.84 × 10^–4^	1.60 × 10^3^	6.26 × 10^–4^	3.90 × 10^10^	2.56 × 10^–11^	6.14 × 10^10^	1.63 × 10^–11^
333	8.43 × 10^3^	1.19 × 10^–4^	1.22 × 10^4^	8.20 × 10^–5^	5.64 × 10^10^	1.77 × 10^–11^	8.16 × 10^10^	1.23 × 10^–11^
373	5.86 × 10^4^	1.71 × 10^–5^	7.90 × 10^4^	1.27 × 10^–5^	7.93 × 10^10^	1.26 × 10^–11^	1.07 × 10^11^	9.35 × 10^–12^
400	1.74 × 10^5^	5.74 × 10^–6^	2.26 × 10^5^	4.42 × 10^–6^	9.61 × 10^10^	1.04 × 10^–11^	1.25 × 10^11^	8.01 × 10^–12^
450	9.26 × 10^5^	1.08 × 10^–6^	1.14 × 10^6^	8.76 × 10^–7^	1.29 × 10^11^	7.73 × 10^–12^	1.60 × 10^11^	6.27 × 10^–12^

In Table 2, lifetime data are shown with (τ_*f*,H_^CVT/μOMT^) and without (τ_*f*,H_^CVT/CAG^) tunneling corrections at various temperatures. Let us consider 298 K, where the forward rate constant *k*_*f*,H_^CVT/μOMG^= 1.60 × 10^3^ s^–1^ and the lifetime of the reactant, τ_*f*,H_^CVT/μOAG^, is 6.26 × 10^–4^ s. The forward rate constant without inclusion of tunneling, *k*_*f*,H_^CVT/CAG^, is 1.02 × 10^3^ s^–1^, and the lifetime of the reactant, τ_*f*,H_^CVT/CAG^, is 9.84 × 10^–4^ s. Table 2 also shows the reverse rate constants of G*C* to GC. Notice that, at 298 K, a reverse *k*_*r*,H_^CVT/μOMG^ = 6.14 × 10^10^ s^–1^ and the lifetime of G*C*, τ_*r*,H_^CVT/μOMG^, is 1.63 × 10^–11^ s. Reverse reaction rate without inclusion of tunneling, *k*_*r*,H_^CVT/CAG^, is 3.90 × 10^10^ s^–1^ and lifetime, τ_*r*,H_^CVT/CAG^, of G*C* is 2.56 × 10^–11^ s.

Our computed rate constants and lifetimes also have ramifications for the KIE and Arrhenius plot analyses shown next.

### KIE and Arrhenius Plots

Figure 6 shows that, for GC → G*C*, KIE = *k*_H_/k_D_ with μOMT increases from 3.19 to 9.10 with decreasing temperatures from 450 to 200 K (red curve). In comparison, KIEs are larger when obtained without tunneling corrections and range from 3.66 to 17.31 also with a decrease of temperatures from 450 to 200 K (blue curve). At 298 K, KIE_μOMT_ = 5.05 and KIE_CVT/CAG_ = 6.85. Our computed KIE_μOMT_ is similar to the experimental KIE of ∼3.5 reported by Al-Hashimi and co-workers^58^ for the wobble and Watson–Crick-like guanine–thymine (GT) mismatches performed in H_2_O and D_2_O. Our KIE_μOMT_ is about 20,000 times smaller than the *c*_OQS_ reported by Slocombe and co-workers,^29^ indicating an importance of multidimensional tunneling, as we highlight here.

**Figure 6:**
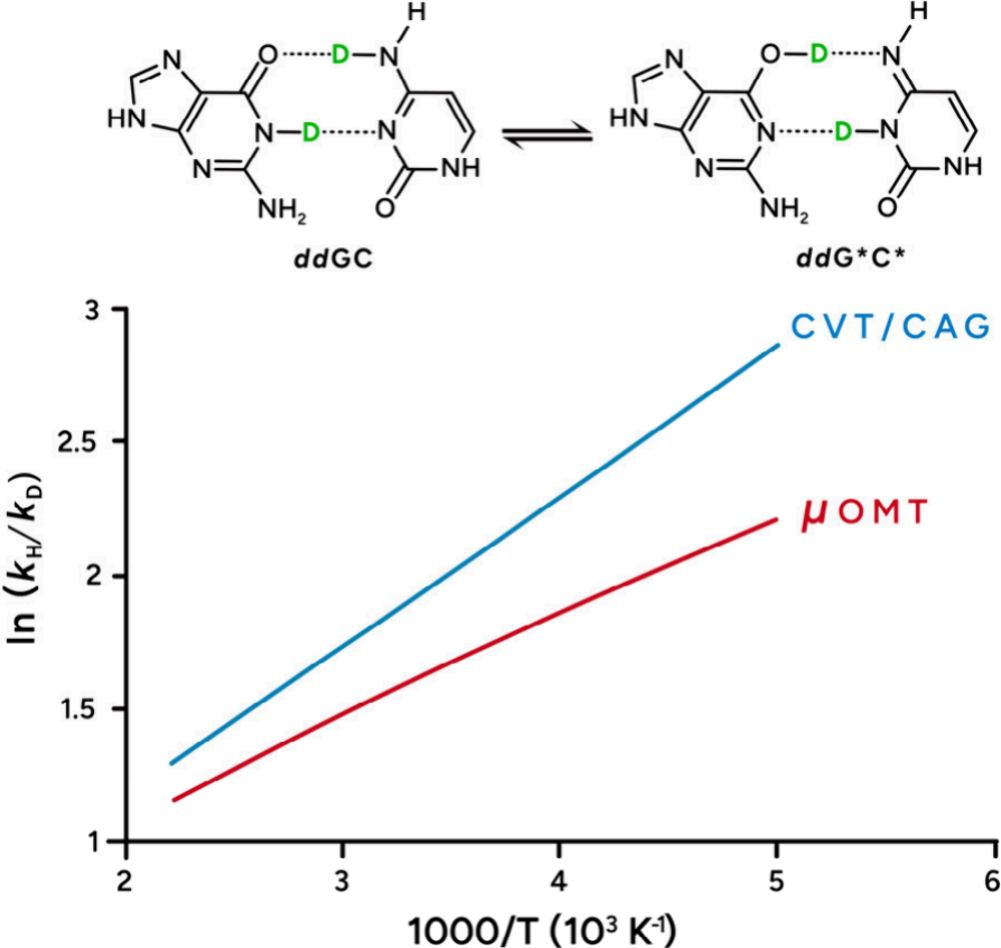
Arrhenius plots of ln­(*k*_H_/*k*_D_) vs 1000/*T* for the forward tautomerization of GC involving the simultaneous transfer of two protons or two deuterons.

Figure 6 reveals that the μOMT trace has a negative curvature indicating a decrease in the tunneling contributions at lower temperatures, which we have observed previously in our studies of THC.^5^ The reason for this negative curvature is due to the presence of the two barriers, overall widening the barrier, as shown in Figure 5. Notice, in Table 1, the κ values for GC and double deuterated GC (*dd*GC). The κ values with tunneling for GC and the κ values with tunneling for *dd*GC are substantially different; i.e., the tunneling contribution is greater with deuterium. This greater tunneling with deuterium is quite unusual but has been seen before.^31,38,59–65^ In our case, the barrier width for the *V*_a_^G^ in *dd*GC is narrower than that for GC (cf. Figure 5 and Figure 7). The larger barrier width is attributed to the zero-point energy associated with the double proton transfer, which decreases in deuterated *dd*GC due to an increase of the mass of the transferred atoms. Another striking feature of Figure 6 is that ln­(KIE) with the inclusion of tunneling is lower than ln­(KIE) when tunneling is not included. *V*_a_^G^ for *k*_H_ represents a thicker tunneling barrier than *V*_a_^G^ for *k*_D_ (cf. Figure 5 and Figure 7); this is due to the ZPE contributing to *V*_a_^G^ through the higher-frequency N–H and O–H stretching modes, as compared to the lower-frequency N–D and O–D stretching modes. These stretching modes originally corresponded to the reaction coordinate but evolved into transverse vibrational modes (Figure S6). Since these new transverse modes have very high frequency, they contribute a substantial amount to the ZPE and give rise to the quantum barrier. The ZPE accumulates over a broad region of the MEP, from about *s* = 0.2 to *s* = 1.0 Å-amu^1/2^. Above, negative curvature is predicted over temperatures from 450 to 200 K. In related work, a low KIE has been attributed to coupling between a neutral N_2_ matrix and the migrating H and D atoms.^66^

**Figure 7:**
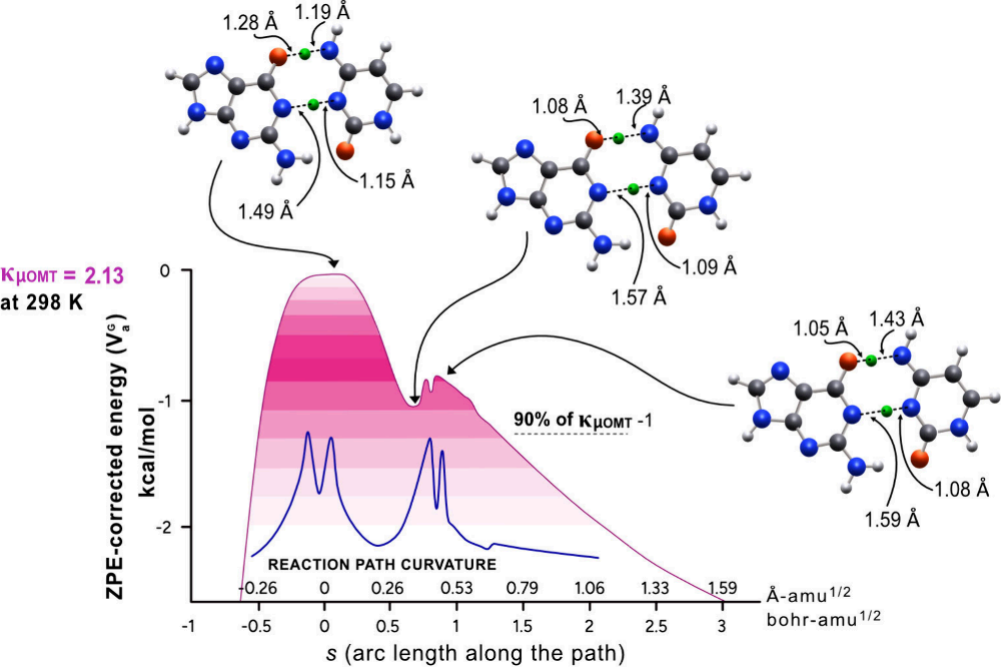
*V*_a_^G^ for double deuterated tautomerization of *dd*GC to *dd*G*C*, where 0 of the energy is the highest point on *V*_a_^G^. Deuterium is marked with a green colored atom.

An experimental test could be carried out on the GC tautomerization with double deuterium substitution, as shown in Figure 6, in which measurements over a decreasing temperature range would yield negative curvature (Figure 6), but importantly, it would not be exponentially increasing.

In summary, double proton transfer in the GC → G*C* tautomerization involves competing quantum effects: tunneling and vibrational ZPE. In GC → G*C*, the ZPE along the reaction path creates a quantum barrier, which was absent on MEP (Figure 2). The quantum barrier greatly increases the width of the full barrier and thereby suppresses tunneling. The quantum barrier appears only in one place along the entire path, where the curvature is large. The curvature is closely related to the force constant matrix, and both are defined in terms of second derivatives.^43^ Since there is a close relation between curvature and force constant matrix, the quantum barrier is only seen where the curvature is high.

The two barriers in Figure 5 create an effective intermediate. The lifetime of the hidden intermediate can be estimated using transition state theory (TST) with an effective “double-TS” model. The intermediate may be observable by using time-resolved experimental techniques.

Analogous changes in *V*_a_^G^ resulting from the interaction of the reaction coordinate with the transverse modes were previously reported in a collinear triatomic system^67,68^ and later in a polyatomic case.^69^ A similar double barrier on *V*_a_^G^ computed for H-transfer has been reported previously.^31,70^ As the path passes through the TS and curves toward the product, the proton motion that was prominent in the transition vector becomes part of the transverse or bound modes which add to the ZPE. With two protons being transferred, there are two high frequency transverse modes that contribute a total of ∼1.0 kcal/mol in GC. While we previously reported a quantum barrier found in a keto–enol tautomerization in the biosynthesis step of THC,^5^ its quantum barrier was smaller than in the present case with GC. With GC, the larger quantum barrier increases the width of the full barrier and reduces the capacity for tunneling. These results highlight the significance of the interplay between tunneling and the zero-point energy (ZPE).

## Conclusion

In the double-proton tautomerization of GC, tunneling is predicted to be strongly suppressed by the height and width of *V*_a_^G^ for the double proton transfer. This barrier consists largely of vibrational ZPE (a quantum barrier). The location of the quantum barrier on the path is dictated by the region of large curvature in the reaction path associated with product formation. As the path swerves from the proton-transfer transition vector toward formation of the product tautomer, the former transition vector evolves into a bound mode with substantial ZPE. This is key to understanding both GC double-proton transfer events that require VTST with multidimensional tunneling (e.g., 3*N* – 6 dimensions, where *N* = 29, not just in one dimension for GC → G*C*). This multidimensional tunneling (μOMT) assessment was essential for deducing the tunneling contributions.

The present work adds to the understanding of tunneling in GC tautomerization, where *V*_a_^G^ is found to have a double barrier, the second of which is a quantum barrier. Understanding of this feature was needed since importantly this double barrier widens the GC → G*C* barrier and lengthens the G*C* lifetime.^71^ The GC → G*C* KIE_μOMT_ is lower than anticipated,^40,72^ only 5.05 at 298 K. Additionally, the Arrhenius plot of ln­(KIE) exhibits a negative curvature over the entire temperature range, indicating that the tunneling contribution decreases at lower temperatures. Nevertheless, the results indicate that tunneling remains relevant in this reaction, even at body temperature. With κ_μ__OMT_ = 1.57, tunneling enhances the rate of GC → G*C* by 36% compared to the variational transition state theory rate. This is a model system in which the sugar–phosphate backbone can constrain the GC pair in a more rigid conformation. Thus, it is conceivable that the barrier width may be reduced in the cellular (condensed) environment compared with the gas phase.

Looking ahead, additional research could also focus on the wobble and Watson–Crick-like GT mismatches^58,73,74^ and elimination reactions, which show lower KIEs than expected.^66^ Using multidimensional tunneling techniques, future studies could investigate whether the quantum barrier plays a significant role in the tautomerization of these mismatches and the lowering of KIEs in eliminations.

## Supplementary Material



## Data Availability

The data underlying this study are available in the published article and its Supporting Information.

## Notes

Gaussian 16 calculated structures and energies of species; POLYRATE calculated κ_CVT/CAG_(*T*), κ_ZCT_(*T*), κ_SCT_(*T*), κ_LCT_(*T*), and κ_μ__OMT_(*T*), rate constants, lifetimes, free energies at 3 temperatures, and scalar curvature analysis for GC → G*C* and *dd*GC → *dd*G*C* using ωB97XD/6-311+G­(d,p); selected frequencies along GC tautomerization with eigenvectors; sample POLYRATE input files (PDF)
