# Supplementary material for: Unexpected Suppression of Double-Proton Tunneling Induced by Quantum Barriers from Zero-Point Energy
Source: J Org Chem. 2025 Jul 17;90(30):10599–606. doi: 10.1021/acs.joc.5c00827 (PMC12322913; doi:10.1021/acs.joc.5c00827)
Supplement: Supplementary file 1 [file jo5c00827_si_001.pdf]

Unexpected Suppression of Double-Proton Tunneling Induced by Quantum Barriers from Zero-Point Energy

Edyta M. Greer,[1*] Florence Uritsky,[1] Benjamin Herrera,[1] Frankie Benavides,[1] Alexander Greer,[2] Charles Doubleday[3*]

[1]Department of Natural Sciences, Baruch College of the City University of New York, New York, New York 10010, United States.
[2]Department of Chemistry, Brooklyn College, Brooklyn, New York 11210, United States; Ph.D. Program in Chemistry, The Graduate Center of the City University of New York, New York 10016, United States
[3]Department of Chemistry, Columbia University, New York, New York 10027, United States

Supporting Information Section

**Figure S1.** Potential energy surface for the tautomerization of GC to G*C* using ωB97XD/6-311+G(d,p)	S3
Calculated structures and energies for the tautomerization of GC to G*C* using ωB97XD/6-311+G(d,p)	S3
**Figure S2.** Potential energy surface for the tautomerization of AT to A*T* using ωB97XD/6-311+G(d,p)	S7
Calculated structures and energies for the tautomerization of AT to A*T* using ωB97XD/6-311+G(d,p)	S7
Reaction energies written by POLYRATE	S10
**Table S1.** Tautomerization of GC to G*C* using ωB97XD/6-311+G(d,p):<br>• transmission coefficients $\kappa_{\mathrm{CVT/CAG}}(T)$, $\kappa_{\mathrm{ZCT}}(T)$, $\kappa_{\mathrm{SCT}}(T)$, $\kappa_{\mathrm{LCT}}(T)$ and $\kappa_{\mu\mathrm{OMT}}(T)$<br>• forward and reverse rate constants<br>**Table S2.** Semi-classical forward rate constants ($k_{f,\mathrm{H}}^{\mathrm{CVT/SCT}}$), μOMT forward tunneling rate constants ($k_{f,\mathrm{H}}^{\mathrm{CVT/\mu OMT}}$) along with lifetimes ($\tau$) for the tautomerization of GC to G*C* using ωB97XD/6-311+G(d,p)	S11<br>S11<br>S12
**Table S3.** Semi-classical reverse rate constants ($k_{r,\mathrm{H}}^{\mathrm{CVT/CAG}}$), μOMT reverse tunneling rate constants ($k_{r,\mathrm{H}}^{\mathrm{CVT/\mu OMT}}$) along with lifetimes ($\tau$) for the tautomerization of GC to G*C* using ωB97XD/6-311+G(d,p)	S13
**Table S4.** Tautomerization of *dd*GC to *dd*G*C* using ωB97XD/6-311+G(d,p):<br>• transmission coefficients $\kappa_{\mathrm{CVT/CAG}}(T)$, $\kappa_{\mathrm{ZCT}}(T)$, $\kappa_{\mathrm{SCT}}(T)$, $\kappa_{\mathrm{LCT}}(T)$ and $\kappa_{\mu\mathrm{OMT}}(T)$<br>• forward and reverse rate constants	S13<br>S14
**Figure S3.** Free energies (kcal/mol) for tautomerization of GC to G*C* at 3 temperatures: 273, 373, and 450K, using ωb97xd/6-311+G(d,p)	S15

**Figure S4**. Scalar curvature (red curve) and V_a^G (blue curve) for the GC → G*C* normalized at s = 0 for comparison. Curvature units are $Å^{-1}$-$amu^{-1/2}$.	S16
**Figure S5**. Scalar curvature (red curve) and V_a^G (blue curve) for the *dd*GC → *dd*G*C* normalized at s = 0 for comparison. Curvature units are $Å^{-1}$-$amu^{-1/2}$.	S16
**Figure S6**. Selected frequencies [cm^{-1}] along GC tautomerization path [Å-$amu^{1/2}$].	S17
Sample input files for POLYRATE calculations:<br>GC.dat<br>GC.70	 S19<br>S24

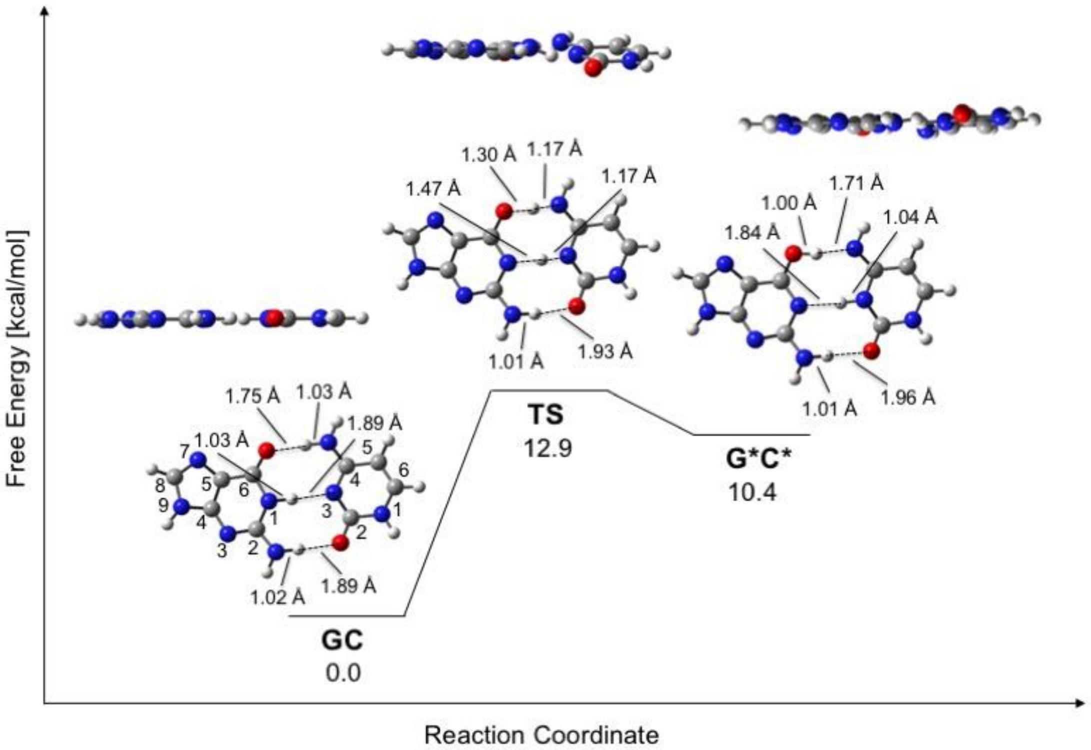

Figure S1. Potential energy surface for the tautomerization of GC to G*C* using ωB97XD/6-311+G(d,p).

Calculated structures and energies for the tautomerization of GC to G*C* via **TS** using ωB97XD/6-311+G(d,p)

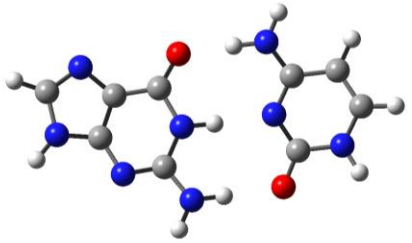

GC

Zero-point correction= 0.220353 (Hartree/Particle)
Thermal correction to Energy= 0.236191
Thermal correction to Enthalpy= 0.237135
Thermal correction to Gibbs Free Energy= 0.175373
Sum of electronic and zero-point Energies= -937.271781
Sum of electronic and thermal Energies= -937.255942
Sum of electronic and thermal Enthalpies= -937.254998
Sum of electronic and thermal Free Energies= -937.316760

E (Thermal) CV S

	KCal/Mol	Cal/Mol-Kelvin	Cal/Mol-Kelvin
Total	148.212	60.780	129.989

0 1

C	-3.25415000	-0.61803800	-0.03243100
C	-2.81137800	0.69758800	-0.05549100
C	-1.40319200	0.92744000	-0.02917800
C	-1.23984100	-1.53214500	0.03644800
C	-4.92564000	0.81596700	-0.10663100
H	0.33499000	-0.18041100	0.03807500
H	-5.94938900	1.15854400	-0.13859700
O	-0.79629500	1.99630700	-0.04215400
N	-0.69165600	-0.27717900	0.01727500
N	-2.53690100	-1.75624100	0.01254300
N	-3.87457000	1.57687500	-0.10184400
N	-4.61553200	-0.52877400	-0.06572200
H	0.63416200	-2.43966600	0.10083400
H	-0.77376700	-3.48606400	0.09578500
N	-0.37777300	-2.56377500	0.08226000
C	2.76510300	1.31056200	0.05452900
C	4.19640800	1.48183100	0.08144200
C	4.94425700	0.36142400	0.12556800
C	2.95936100	-1.01846000	0.11629700
H	4.65047300	2.46192400	0.06706200
H	6.02662700	0.37368600	0.14890700
N	2.20364200	0.10404900	0.07230700
H	2.34898000	3.29697800	-0.00442100
H	0.93626900	2.24817200	-0.00915500
N	1.96220700	2.37022300	0.01088100
N	4.35010400	-0.85823000	0.14273200
O	2.50079000	-2.15471800	0.13412100
H	4.89038300	-1.70835200	0.17586400
H	-5.25237500	-1.30750200	-0.06048200

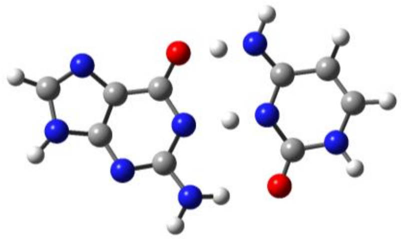

TS

Zero-point correction= 0.213410
(Hartree/Particle)
Thermal correction to Energy= 0.228395
Thermal correction to Enthalpy= 0.229339
Thermal correction to Gibbs Free Energy= 0.170304
Sum of electronic and zero-point Energies= -937.253159
Sum of electronic and thermal Energies= -937.238173
Sum of electronic and thermal Enthalpies= -937.237229
Sum of electronic and thermal Free Energies= -937.296265

E (Thermal)	CV	S

	KCal/Mol	Cal/Mol-Kelvin	Cal/Mol-Kelvin
Total	143.317	58.662	124.255

0 1

C	-3.14222700	-0.39749200	0.00686000
C	-2.66172000	0.88831400	-0.18871100
C	-1.25348800	1.05505700	-0.16837900
C	-1.12841400	-1.28394200	0.20589100
C	-4.77214800	1.05826800	-0.26604500
H	0.94660800	0.05685400	-0.02686400
H	-5.78599900	1.42191400	-0.34975100
O	-0.68416900	2.17558600	-0.31264400
N	-0.51715400	-0.08201900	0.02377700
N	-2.43519600	-1.51433700	0.20627900
N	-3.70165400	1.78573400	-0.35558500
N	-4.50320400	-0.27877200	-0.04570200
H	0.64952700	-2.31671300	0.13993200
H	-0.77680900	-3.24488800	0.39914500
N	-0.31583400	-2.35216800	0.44572200
C	2.57637200	1.49280900	0.13403800
C	4.00406900	1.68349400	0.23225400
C	4.79970600	0.60714800	0.10986200
C	2.90223600	-0.87032500	-0.16871900
H	4.41211200	2.67122400	0.38671100
H	5.87940600	0.66486800	0.16135600
N	2.09993900	0.22926500	-0.04227200
H	2.09381200	3.39594000	0.32870600
H	0.57684400	2.30786900	-0.02606700
N	1.71810800	2.46897800	0.20109700
N	4.27147200	-0.63061800	-0.09462800
O	2.48919800	-2.00081900	-0.33464800
H	4.85616600	-1.44633400	-0.18571300
H	-5.16361400	-1.02953000	0.06178300

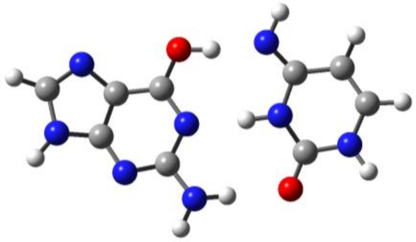

G*C*

Zero-point correction= (Hartree/Particle)	0.220305
Thermal correction to Energy=	0.235905
Thermal correction to Enthalpy=	0.236849
Thermal correction to Gibbs Free Energy=	0.175793
Sum of electronic and zero-point Energies=	-937.255759
Sum of electronic and thermal Energies=	-937.240159
Sum of electronic and thermal Enthalpies=	-937.239215
Sum of electronic and thermal Free Energies=	-937.300271

E (Thermal)	CV	S
KCal/Mol	Cal/Mol-Kelvin	Cal/Mol-Kelvin

Total	148.032	60.084	128.503

0 1

C	-3.25071400	-0.60967800	-0.02774300
C	-2.79205300	0.70273700	-0.08336800
C	-1.39741000	0.85620200	-0.02437300
C	-1.21564300	-1.45868100	0.12949400
C	-4.90064900	0.84094400	-0.19716000
H	1.20510200	-0.10621000	0.07127600
H	-5.91937700	1.19332500	-0.26912800
O	-0.86639900	2.04990400	-0.07003700
N	-0.62843200	-0.23216400	0.07753800
N	-2.52224400	-1.71802400	0.07587300
N	-3.84556400	1.59470900	-0.18907200
N	-4.61209900	-0.50717700	-0.10270200
H	0.61524100	-2.43287000	0.15644600
H	-0.80600800	-3.43104700	0.19559900
N	-0.38725700	-2.52143300	0.27086400
C	2.69835900	1.33710300	0.09934100
C	4.14476500	1.48883000	0.10996100
C	4.91878500	0.39444100	0.07632900
C	3.01069400	-1.09628100	0.02912600
H	4.57742600	2.47797800	0.14155300
H	6.00018000	0.44097600	0.07965800
N	2.24075800	0.02860100	0.06513800
H	2.23896300	3.19314200	0.14222900
H	0.13098900	2.04907900	0.00882600
N	1.81956100	2.27093000	0.11643200
N	4.37664000	-0.86416700	0.03357700
O	2.56105300	-2.22612400	-0.00438300
H	4.95616500	-1.68720500	0.01037500
H	-5.26196400	-1.27459100	-0.08658700

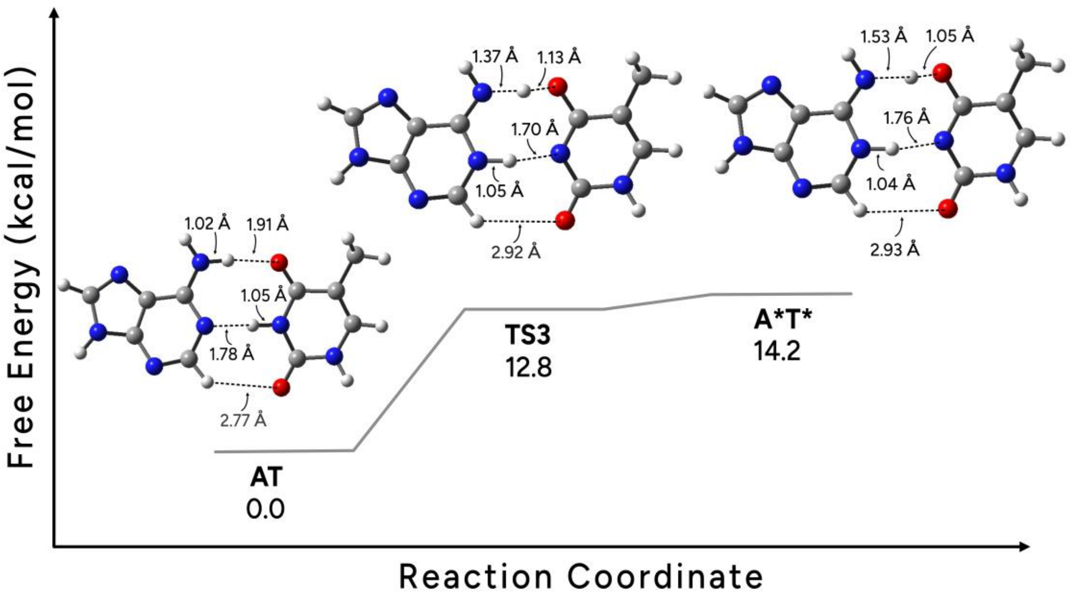

Figure S2. Potential energy surface for the tautomerization of AT to A*T* using ωB97XD/6-311+G(d,p).

Calculated structures and energies for the tautomerization of AT to A*T* via **TS** using ωB97XD/6-311+G(d,p)

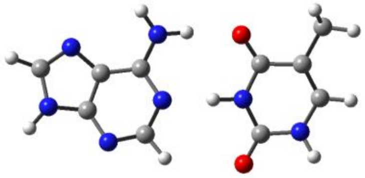

AT

Zero-point correction= 0.230911
(Hartree/Particle)
Thermal correction to Energy= 0.247367
Thermal correction to Enthalpy= 0.248311
Thermal correction to Gibbs Free Energy= 0.184648
Sum of electronic and zero-point Energies= -921.205549
Sum of electronic and thermal Energies= -921.189094
Sum of electronic and thermal Enthalpies= -921.188149
Sum of electronic and thermal Free Energies= -921.251812

	E (Thermal)	CV	S
	KCal/Mol	Cal/Mol-Kelvin	Cal/Mol-Kelvin
Total	155.225	61.516	133.990

0 1

C	-3.07792400	0.57667700	-0.00016800
C	-3.54752700	-0.72855100	-0.00008200
C	-1.52126300	-1.57900000	0.00009200
C	-1.67846100	0.74045300	-0.00011100
C	-5.18617100	0.74200600	-0.00029300

H	-0.83724300	-2.42312500	0.00019800
H	-6.20115700	1.11164400	-0.00037700
C	2.51310800	0.90040800	0.00003100
C	2.52186300	-1.56557600	0.00026600
C	4.59380900	-0.29857400	0.00018300
H	5.67355000	-0.39073000	0.00021000
O	1.84286200	1.92541000	-0.00008000
O	1.92951900	-2.62242900	0.00033500
N	-0.92753000	-0.37461200	0.00002100
N	-2.81618700	-1.84846200	0.00004900
N	-4.91238600	-0.60467900	-0.00016200
N	-4.11701000	1.48512500	-0.00029400
N	1.90252700	-0.33868200	0.00013300
N	3.90381700	-1.48625400	0.00027800
H	0.85732000	-0.35458700	0.00011200
C	3.97608300	0.89565900	0.00006300
C	4.69235200	2.20967200	-0.00004400
H	4.41737000	2.79941900	-0.87789100
H	4.41733500	2.79958100	0.87768400
H	5.77470700	2.06555600	-0.00000800
H	-0.06732700	2.01161000	-0.00013200
N	-1.08417400	1.93850000	-0.00017900
H	-1.65645400	2.76466400	-0.00027600
H	-5.57318500	-1.36362200	-0.00013300
H	4.38838200	-2.36885200	0.00036400

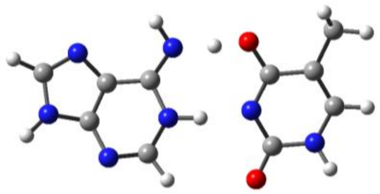

TS

Zero-point correction= (Hartree/Particle)	0.226860
Thermal correction to Energy=	0.242289
Thermal correction to Enthalpy=	0.243234
Thermal correction to Gibbs Free Energy=	0.182470
Sum of electronic and zero-point Energies=	-921.187063
Sum of electronic and thermal Energies=	-921.171634
Sum of electronic and thermal Enthalpies=	-921.170689
Sum of electronic and thermal Free Energies=	-921.231453

	E (Thermal) KCal/Mol	CV Cal/Mol-Kelvin	S Cal/Mol-Kelvin
Total	152.039	58.770	127.888

0 1

C	-2.91255100	0.77787300	-0.00018300
C	-3.48410000	-0.48048500	-0.00009200
C	-1.56322300	-1.54872700	0.00007800
C	-1.48811200	0.87270500	-0.00013700
C	-5.00070500	1.11066000	-0.00029300
H	-0.92498300	-2.42623800	0.00018200
H	-5.98224700	1.56057700	-0.00037100

C	2.44108700	1.05516000	-0.00000400
C	2.52846700	-1.30065600	0.00021200
C	4.56583900	0.01080800	0.00017100
H	5.64886100	-0.03180000	0.00021600
O	1.74869500	2.13646000	-0.00012700
O	1.99550800	-2.39820700	0.00029000
N	-0.89457700	-0.37274600	-0.00000200
N	-2.85885400	-1.68209300	0.00004100
N	-4.83107800	-0.25197300	-0.00016300
N	-3.87153600	1.76199100	-0.00030800
N	1.83635200	-0.12546000	0.00008000
N	3.91899100	-1.19044400	0.00025400
H	0.15852800	-0.37583600	0.00003900
C	3.88552600	1.17606100	0.00004000
C	4.53446700	2.52568700	-0.00005700
H	4.23372800	3.10228600	-0.87819700
H	4.23366100	3.10244500	0.87795600
H	5.62290600	2.43389200	-0.00000700
H	0.62331400	1.99449500	-0.00015700
N	-0.74608500	1.93707000	-0.00020500
H	-1.29744200	2.78664200	-0.00030200
H	-5.54726300	-0.95942600	-0.00012700
H	4.43047600	-2.05831100	0.00035200

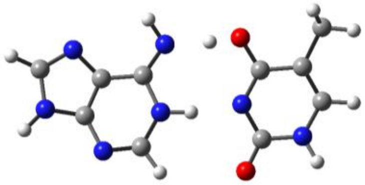

A*T*

Zero-point correction= (Hartree/Particle)	0.230003
Thermal correction to Energy=	0.245875
Thermal correction to Enthalpy=	0.246819
Thermal correction to Gibbs Free Energy=	0.184947
Sum of electronic and zero-point Energies=	-921.184172
Sum of electronic and thermal Energies=	-921.168301
Sum of electronic and thermal Enthalpies=	-921.167356
Sum of electronic and thermal Free Energies=	-921.229229

	E (Thermal) KCal/Mol	CV Cal/Mol-Kelvin	S Cal/Mol-Kelvin
Total	154.289	59.851	130.222

0 1

C	-2.93866300	0.77326500	-0.00023700
C	-3.50356500	-0.48691800	-0.00014000
C	-1.57926400	-1.54920300	0.00047100
C	-1.51078300	0.88157400	0.00007100
C	-5.02966900	1.09480600	-0.00077500
H	-0.93810200	-2.42460000	0.00075300
H	-6.01354700	1.53954600	-0.00108000
C	2.48595600	1.06143200	0.00031500
C	2.54017500	-1.28571100	0.00027900

C	4.59396900	0.00123500	-0.00025800
H	5.67631900	-0.05377200	-0.00057200
O	1.80745800	2.16507700	0.00052800
O	1.99498000	-2.37522600	0.00003600
N	-0.91566800	-0.37039300	0.00043400
N	-2.87349800	-1.68831000	0.00021000
N	-4.85222800	-0.26648100	-0.00049200
N	-3.90327000	1.75162100	-0.00061900
N	1.86133300	-0.09987300	0.00039500
N	3.93261100	-1.19044500	-0.00019700
H	0.12662100	-0.37167100	0.00064600
C	3.92681500	1.17530300	0.00003200
C	4.59042300	2.51814000	-0.00001900
H	4.29712500	3.09812000	-0.87834100
H	4.29754400	3.09798200	0.87853500
H	5.67748800	2.41310100	-0.00028600
H	0.76396300	2.02113600	0.00061900
N	-0.76720600	1.93876600	0.00007500
H	-1.33301200	2.78040700	-0.00021100
H	-5.56393900	-0.97836300	-0.00053500
H	4.43408700	-2.06453300	-0.00045600

Reaction energetics written by POLYRATE

Tautomerization of GC to G*C* using ωB97XD/6-311+G(d,p)

Saddle point energetics (V = classical energy, ZPE = zero point energy)

	hartrees	eV	cm**-1	kcal
V w/re reactants V	0.02556	0.69566	5610.86	16.0422
V w/re product V	0.00950	0.25838	2083.98	5.9584
V+ZPE w/re reactant V	0.23897	6.50283	52448.43	149.9576
V+ZPE w/re product V	0.22290	6.06555	48921.55	139.8737
V+ZPE w/re reactant V+ZPE	0.01862	0.50668	4086.61	11.6842
V+ZPE w/re product V+ZPE	0.00260	0.07070	570.25	1.6304
V+ZPE w/re saddle point V	0.21341	5.80717	46837.57	133.9153

Tautomerization of *dd*GC to *dd*G*C* using ωB97XD/6-311+G(d,p)

Saddle point energetics (V = classical energy, ZPE = zero point energy)

	hartrees	eV	cm**-1	kcal
V w/re reactants V	0.02556	0.69566	5610.86	16.0422
V w/re product V	0.00950	0.25838	2083.98	5.9584
V+ZPE w/re reactant V	0.23428	6.37510	51418.26	147.0122
V+ZPE w/re product V	0.21821	5.93782	47891.38	136.9283
V+ZPE w/re reactant V+ZPE	0.02095	0.57001	4597.39	13.1446
V+ZPE w/re product V+ZPE	0.00485	0.13195	1064.25	3.0428
V+ZPE w/re saddle point V	0.20871	5.67944	45807.40	130.9699

For tautomerization of GC to G*C* using ωB97XD/6-311+G(d,p) the following information is given:

- transmission coefficients $\kappa_{CVT/CAG}(T)$, $\kappa_{ZCT}(T)$, $\kappa_{SCT}(T)$, $\kappa_{LCT}(T)$ and $\kappa_{\mu OMT}(T)$
- forward and reverse rate constants

Table S1. Tautomerization of GC to G*C* using ωB97XD/6-311+G(d,p)

Transmission coefficients $\kappa_{CVT/CAG}(T)$, $\kappa_{ZCT}(T)$, $\kappa_{SCT}(T)$, $\kappa_{LCT}(T)$ and $\kappa_{\mu OMT}(T)$

T(K)	CVT/CAG	ZCT	SCT	LCT	μOMT
200.00	4.1602E-01	1.3898E+00	2.1456E+00	2.4043E+00	2.5193E+00
225.00	4.5515E-01	1.2992E+00	1.8423E+00	2.0473E+00	2.1218E+00
250.00	4.8914E-01	1.2374E+00	1.6484E+00	1.8141E+00	1.8662E+00
273.15	5.1681E-01	1.1961E+00	1.5245E+00	1.6628E+00	1.7021E+00
293.00	5.3800E-01	1.1688E+00	1.4453E+00	1.5651E+00	1.5968E+00
298.15	5.4316E-01	1.1626E+00	1.4278E+00	1.5433E+00	1.5735E+00
308.15	5.5280E-01	1.1517E+00	1.3967E+00	1.5047E+00	1.5320E+00
313.00	5.5731E-01	1.1467E+00	1.3828E+00	1.4874E+00	1.5136E+00
323.00	5.6627E-01	1.1373E+00	1.3566E+00	1.4546E+00	1.4785E+00
333.00	5.7482E-01	1.1288E+00	1.3330E+00	1.4251E+00	1.4470E+00
343.00	5.8296E-01	1.1211E+00	1.3117E+00	1.3984E+00	1.4186E+00
353.00	5.9074E-01	1.1140E+00	1.2924E+00	1.3742E+00	1.3929E+00
363.15	5.9827E-01	1.1075E+00	1.2747E+00	1.3519E+00	1.3692E+00
373.00	6.0526E-01	1.1017E+00	1.2590E+00	1.3321E+00	1.3482E+00
383.15	6.1215E-01	1.0961E+00	1.2443E+00	1.3134E+00	1.3285E+00
400.00	6.2292E-01	1.0880E+00	1.2225E+00	1.2858E+00	1.2993E+00
413.15	6.3080E-01	1.0823E+00	1.2075E+00	1.2668E+00	1.2792E+00
438.15	6.3080E-01	1.0729E+00	1.1829E+00	1.2355E+00	1.2462E+00
450.00	6.5069E-01	1.0690E+00	1.1728E+00	1.2226E+00	1.2326E+00

Summary of forward rate constants (s-1):

T(K)	CVT/CAG	CVT/ZCT	CVT/SCT	CVT/LCT	CVT/μOMT
200.00	4.9148E-02	6.8308E-02	1.0545E-01	1.1817E-01	1.2382E-01
225.00	1.4093E+00	1.8309E+00	2.5962E+00	2.8851E+00	2.9902E+00
250.00	2.0622E+01	2.5518E+01	3.3994E+01	3.7410E+01	3.8485E+01
273.15	1.5952E+02	1.9080E+02	2.4320E+02	2.6525E+02	2.7152E+02
293.00	7.1207E+02	8.3225E+02	1.0292E+03	1.1144E+03	1.1370E+03
298.15	1.0160E+03	1.1813E+03	1.4507E+03	1.5681E+03	1.5987E+03
308.15	1.9583E+03	2.2553E+03	2.7351E+03	2.9466E+03	3.0002E+03
313.00	2.6516E+03	3.0407E+03	3.6668E+03	3.9441E+03	4.0134E+03
323.00	4.8128E+03	5.4738E+03	6.5289E+03	7.0008E+03	7.1158E+03
333.00	8.4272E+03	9.5127E+03	1.1233E+04	1.2009E+04	1.2194E+04
343.00	1.4280E+04	1.6009E+04	1.8731E+04	1.9969E+04	2.0258E+04
353.00	2.3483E+04	2.6161E+04	3.0351E+04	3.2270E+04	3.2709E+04
363.15	3.7829E+04	4.1894E+04	4.8221E+04	5.1139E+04	5.1794E+04
373.00	5.8607E+04	6.4565E+04	7.3789E+04	7.8070E+04	7.9015E+04
383.15	8.9871E+04	9.8512E+04	1.1183E+05	1.1804E+05	1.1939E+05
400.00	1.7417E+05	1.8949E+05	2.1293E+05	2.2396E+05	2.2630E+05
413.15	2.8115E+05	3.0427E+05	3.3948E+05	3.5615E+05	3.5963E+05

438.15	6.4535E+05	6.9240E+05	7.6341E+05	7.9736E+05	8.0425E+05
450.00	9.2647E+05	9.9040E+05	1.0866E+06	1.1327E+06	1.1420E+06

Summary of reverse rate constants (s-1):

T(K)	CVT/CAG	CVT/ZCT	CVT/SCT	CVT/LCT	CVT/μOMT
200.00	7.1257E+09	9.9036E+09	1.5289E+10	1.7132E+10	1.7951E+10
225.00	1.2604E+10	1.6375E+10	2.3220E+10	2.5803E+10	2.6743E+10
250.00	1.9937E+10	2.4670E+10	3.2865E+10	3.6167E+10	3.7206E+10
273.15	2.8343E+10	3.3900E+10	4.3211E+10	4.7129E+10	4.8242E+10
293.00	3.6705E+10	4.2899E+10	5.3050E+10	5.7445E+10	5.8609E+10
298.15	3.9037E+10	4.5386E+10	5.5737E+10	6.0247E+10	6.1423E+10
308.15	4.3746E+10	5.0381E+10	6.1100E+10	6.5823E+10	6.7020E+10
313.00	4.6113E+10	5.2880E+10	6.3767E+10	6.8590E+10	6.9795E+10
323.00	5.1158E+10	5.8183E+10	6.9398E+10	7.4414E+10	7.5637E+10
333.00	5.6412E+10	6.3678E+10	7.5194E+10	8.0391E+10	8.1628E+10
343.00	6.1863E+10	6.9353E+10	8.1145E+10	8.6508E+10	8.7759E+10
353.00	6.7499E+10	7.5195E+10	8.7238E+10	9.2756E+10	9.4017E+10
363.15	7.3395E+10	8.1283E+10	9.3558E+10	9.9219E+10	1.0049E+11
373.00	7.9275E+10	8.7334E+10	9.9811E+10	1.0560E+11	1.0688E+11
383.15	8.5485E+10	9.3704E+10	1.0637E+11	1.1228E+11	1.1356E+11
400.00	9.6101E+10	1.0455E+11	1.1748E+11	1.2357E+11	1.2486E+11
413.15	1.0463E+11	1.1323E+11	1.2634E+11	1.3254E+11	1.3383E+11
438.15	1.2133E+11	1.3017E+11	1.4352E+11	1.4991E+11	1.5120E+11
450.00	1.2944E+11	1.3837E+11	1.5181E+11	1.5826E+11	1.5956E+11

Table S2. Semi-classical forward rate constants ($k_{f,\mathrm{H}}^{\mathrm{CVT/SCT}}$), μOMT forward tunneling rate constants ($k_{f,\mathrm{H}}^{\mathrm{CVT}/\mu\mathrm{OMT}}$) along with lifetimes ($\tau$) for the tautomerization of GC to G*C* using ωB97XD/6-311+G(d,p)

T [K]	$k_{f,\mathrm{H}}^{\mathrm{CVT/SCT}}$ [s^{-1}]	$\tau_{f,\mathrm{H}}^{\mathrm{CVT/CAG}}$ [s]	$k_{f,\mathrm{H}}^{\mathrm{CVT}/\mu\mathrm{OMT}}$ [s^{-1}]	$\tau_{f,\mathrm{H}}^{\mathrm{CVT}/\mu\mathrm{OMT}}$ [s]
200	4.91×10^{-2}	2.03×10^{1}	1.24×10^{-1}	8.08
225	1.41	7.10×10^{-1}	2.99	3.34×10^{-1}
250	2.06×10^{1}	4.85×10^{-2}	3.85×10^{1}	2.60×10^{-2}
273.15	1.60×10^{2}	6.27×10^{-3}	2.72×10^{2}	3.68×10^{-3}
293	7.12×10^{2}	1.40×10^{-3}	1.14×10^{3}	8.80×10^{-4}
298.15	1.02×10^{3}	9.84×10^{-4}	1.60×10^{3}	6.26×10^{-4}
308.15	1.96×10^{3}	5.11×10^{-4}	3.00×10^{3}	3.33×10^{-4}
313	2.65×10^{3}	3.77×10^{-4}	4.01×10^{3}	2.49×10^{-4}
323	4.81×10^{3}	2.08×10^{-4}	7.12×10^{3}	1.41×10^{-4}
333	8.43×10^{3}	1.19×10^{-4}	1.22×10^{4}	8.20×10^{-5}
343	1.43×10^{4}	7.00×10^{-5}	2.03×10^{4}	4.94×10^{-5}
353	2.35×10^{4}	4.26×10^{-5}	3.27×10^{4}	3.06×10^{-5}
363.15	3.78×10^{4}	2.64×10^{-5}	5.18×10^{4}	1.93×10^{-5}
373	5.86×10^{4}	1.71×10^{-5}	7.90×10^{4}	1.27×10^{-5}
383.15	8.99×10^{4}	1.11×10^{-5}	1.19×10^{5}	8.38×10^{-6}
400	1.74×10^{5}	5.74×10^{-6}	2.26×10^{5}	4.42×10^{-6}
413.15	2.81×10^{5}	3.56×10^{-6}	3.60×10^{5}	2.78×10^{-6}

438.15	6.45×10^{5}	1.55×10^{-6}	8.04×10^{5}	1.24×10^{-6}
450	9.26×10^{5}	1.08×10^{-6}	1.14×10^{6}	8.76×10^{-7}

Table S3. Semi-classical reverse rate constants ($k_{r,\mathrm{H}}^{\mathrm{CVT/CAG}}$), μOMT reverse tunneling rate constants ($k_{r,\mathrm{H}}^{\mathrm{CVT}/\mu\mathrm{OMT}}$) along with lifetimes ($\tau$) for the tautomerization of GC to G*C* using ωB97XD/6-311+G(d,p)

T [K]	$k_{r,\mathrm{H}}^{\mathrm{CVT/SCT}}$ [s^{-1}]	$\tau_{r,\mathrm{H}}^{\mathrm{CVT/CAG}}$ [s]	$k_{r,\mathrm{H}}^{\mathrm{CVT}/\mu\mathrm{OMT}}$ [s^{-1}]	$\tau_{r,\mathrm{H}}^{\mathrm{CVT}/\mu\mathrm{OMT}}$ [s]
200	7.13×10^{9}	1.40×10^{-10}	1.80×10^{10}	5.57×10^{-11}
225	1.26×10^{10}	7.93×10^{-11}	2.67×10^{10}	3.74×10^{-11}
250	1.99×10^{10}	5.02×10^{-11}	3.72×10^{10}	2.69×10^{-11}
273.15	2.83×10^{10}	3.53×10^{-11}	4.82×10^{10}	2.07×10^{-11}
293	3.67×10^{10}	2.72×10^{-11}	5.86×10^{10}	1.71×10^{-11}
298.15	3.90×10^{10}	2.56×10^{-11}	6.14×10^{10}	1.63×10^{-11}
308.15	4.37×10^{10}	2.29×10^{-11}	6.70×10^{10}	1.49×10^{-11}
313	4.61×10^{10}	2.17×10^{-11}	6.98×10^{10}	1.43×10^{-11}
323	5.12×10^{10}	1.95×10^{-11}	7.56×10^{10}	1.32×10^{-11}
333	5.64×10^{10}	1.77×10^{-11}	8.16×10^{10}	1.23×10^{-11}
343	6.19×10^{10}	1.62×10^{-11}	8.78×10^{10}	1.14×10^{-11}
353	6.75×10^{10}	1.48×10^{-11}	9.40×10^{10}	1.06×10^{-11}
363.15	7.34×10^{10}	1.36×10^{-11}	1.00×10^{11}	9.95×10^{-12}
373	7.93×10^{10}	1.26×10^{-11}	1.07×10^{11}	9.36×10^{-12}
383.15	8.55×10^{10}	1.17×10^{-11}	1.14×10^{11}	8.81×10^{-12}
400	9.61×10^{10}	1.04×10^{-11}	1.25×10^{11}	8.01×10^{-12}
413.15	1.05×10^{11}	9.56×10^{-12}	1.34×10^{11}	7.47×10^{-12}
438.15	1.21×10^{11}	8.24×10^{-12}	1.51×10^{11}	6.61×10^{-12}
450	1.29×10^{11}	7.73×10^{-12}	1.60×10^{11}	6.27×10^{-12}

For tautomerization of *dd*GC to *dd*G*C* using ωB97XD/6-311+G(d,p) the following information is given:

- transmission coefficients $\kappa_{\mathrm{CVT/CAG}}(T)$, $\kappa_{\mathrm{ZCT}}(T)$, $\kappa_{\mathrm{SCT}}(T)$, $\kappa_{\mathrm{LCT}}(T)$ and $\kappa_{\mu\mathrm{OMT}}(T)$
- forward and reverse rate constants

Table S4. Tautomerization of *dd*GC to *dd*G*C* using ωB97XD/6-311+G(d,p)

Transmission coefficients $\kappa_{\mathrm{CVT/CAG}}(T)$, $\kappa_{\mathrm{ZCT}}(T)$, $\kappa_{\mathrm{SCT}}(T)$, $\kappa_{\mathrm{LCT}}(T)$ and $\kappa_{\mathrm{muOMT}}(T)$

T(K)	CVT/CAG	ZCT	SCT	LCT	μOMT
200.00	9.1703E-01	1.6653E+00	4.7941E+00	2.6692E+00	4.7941E+00
225.00	9.1671E-01	1.4918E+00	3.5572E+00	2.1500E+00	3.5572E+00
250.00	9.1637E-01	1.3801E+00	2.8527E+00	1.8458E+00	2.8527E+00
273.15	9.1612E-01	1.3083E+00	2.4382E+00	1.6634E+00	2.4382E+00
293.00	9.1589E-01	1.2622E+00	2.1879E+00	1.5516E+00	2.1879E+00
298.15	9.1582E-01	1.2520E+00	2.1341E+00	1.5275E+00	2.1341E+00

308.15	9.1569E-01	1.2338E+00	2.0400E+00	1.4850E+00	2.0400E+00
313.00	9.1562E-01	1.2257E+00	1.9987E+00	1.4663E+00	1.9987E+00
323.00	9.1547E-01	1.2104E+00	1.9212E+00	1.4312E+00	1.9212E+00
333.00	9.1531E-01	1.1966E+00	1.8528E+00	1.4000E+00	1.8528E+00
343.00	9.1513E-01	1.1841E+00	1.7920E+00	1.3722E+00	1.7920E+00
353.00	9.1494E-01	1.1728E+00	1.7378E+00	1.3474E+00	1.7378E+00
363.15	9.1471E-01	1.1624E+00	1.6884E+00	1.3247E+00	1.6884E+00
373.00	9.1447E-01	1.1532E+00	1.6453E+00	1.3048E+00	1.6453E+00
383.15	9.1421E-01	1.1446E+00	1.6051E+00	1.2862E+00	1.6051E+00
400.00	9.1373E-01	1.1317E+00	1.5465E+00	1.2590E+00	1.5465E+00
413.15	9.1332E-01	1.1229E+00	1.5066E+00	1.2405E+00	1.5066E+00
438.15	9.1249E-01	1.1084E+00	1.4423E+00	1.2105E+00	1.4423E+00
450.00	9.1206E-01	1.1025E+00	1.4162E+00	1.1982E+00	1.4162E+00

Summary of forward rate constants (s-1):

T(K)	CVT/CAG	CVT/ZCT	CVT/SCT	CVT/LCT	CVT/μOMT
200.00	2.8391E-03	4.7281E-03	3.0960E-03	7.5782E-03	1.3611E-02
225.00	1.1168E-01	1.6661E-01	1.2183E-01	2.4011E-01	3.9727E-01
250.00	2.0996E+00	2.8976E+00	2.2912E+00	3.8756E+00	5.9895E+00
273.15	1.9630E+01	2.5681E+01	2.1427E+01	3.2651E+01	4.7861E+01
293.00	1.0057E+02	1.2694E+02	1.0981E+02	1.5605E+02	2.2005E+02
298.15	1.4827E+02	1.8563E+02	1.6190E+02	2.2648E+02	3.1643E+02
308.15	3.0354E+02	3.7451E+02	3.3148E+02	4.5075E+02	6.1921E+02
313.00	4.2260E+02	5.1799E+02	4.6154E+02	6.1966E+02	8.4463E+02
323.00	8.1014E+02	9.8058E+02	8.8495E+02	1.1595E+03	1.5565E+03
333.00	1.4933E+03	1.7869E+03	1.6315E+03	2.0907E+03	2.7668E+03
343.00	2.6558E+03	3.1448E+03	2.9021E+03	3.6444E+03	4.7593E+03
353.00	4.5713E+03	5.3613E+03	4.9963E+03	6.1591E+03	7.9438E+03
363.15	7.6930E+03	8.9426E+03	8.4103E+03	1.0191E+04	1.2989E+04
373.00	1.2407E+04	1.4308E+04	1.3568E+04	1.6189E+04	2.0414E+04
383.15	1.9788E+04	2.2649E+04	2.1645E+04	2.5451E+04	3.1762E+04
400.00	4.0759E+04	4.6128E+04	4.4607E+04	5.1315E+04	6.3031E+04
413.15	6.8762E+04	7.7213E+04	7.5288E+04	8.5297E+04	1.0360E+05
438.15	1.7047E+05	1.8895E+05	1.8681E+05	2.0634E+05	2.4587E+05
450.00	2.5309E+05	2.7902E+05	2.7749E+05	3.0326E+05	3.5842E+05

Summary of reverse rate constants (s-1):

T(K)	CVT/CAG	CVT/ZCT	CVT/SCT	CVT/LCT	CVT/μOMT
200.00	4.7111E+08	7.8455E+08	2.2585E+09	1.2575E+09	2.2585E+09
225.00	1.1318E+09	1.6884E+09	4.0260E+09	2.4333E+09	4.0260E+09
250.00	2.2832E+09	3.1509E+09	6.5131E+09	4.2143E+09	6.5131E+09
273.15	3.9019E+09	5.1047E+09	9.5137E+09	6.4902E+09	9.5137E+09
293.00	5.7778E+09	7.2927E+09	1.2641E+10	8.9651E+09	1.2641E+10
298.15	6.3434E+09	7.9418E+09	1.3538E+10	9.6893E+09	1.3538E+10
308.15	7.5379E+09	9.3004E+09	1.5377E+10	1.1194E+10	1.5377E+10
313.00	8.1637E+09	1.0007E+10	1.6316E+10	1.1971E+10	1.6316E+10
323.00	9.5513E+09	1.1561E+10	1.8350E+10	1.3670E+10	1.8350E+10
333.00	1.1072E+10	1.3248E+10	2.0513E+10	1.5500E+10	2.0513E+10
343.00	1.2725E+10	1.5068E+10	2.2804E+10	1.7462E+10	2.2804E+10
353.00	1.4514E+10	1.7022E+10	2.5221E+10	1.9555E+10	2.5221E+10
363.15	1.6466E+10	1.9141E+10	2.7802E+10	2.1812E+10	2.7802E+10
373.00	1.8492E+10	2.1326E+10	3.0425E+10	2.4128E+10	3.0425E+10
383.15	2.0715E+10	2.3709E+10	3.3249E+10	2.6643E+10	3.3249E+10

400.00	2.4702E+10	2.7956E+10	3.8200E+10	3.1099E+10	3.8200E+10
413.15	2.8067E+10	3.1516E+10	4.2286E+10	3.4816E+10	4.2286E+10
438.15	3.5057E+10	3.8858E+10	5.0564E+10	4.2435E+10	5.0564E+10
450.00	3.8633E+10	4.2591E+10	5.4711E+10	4.6291E+10	5.4711E+10

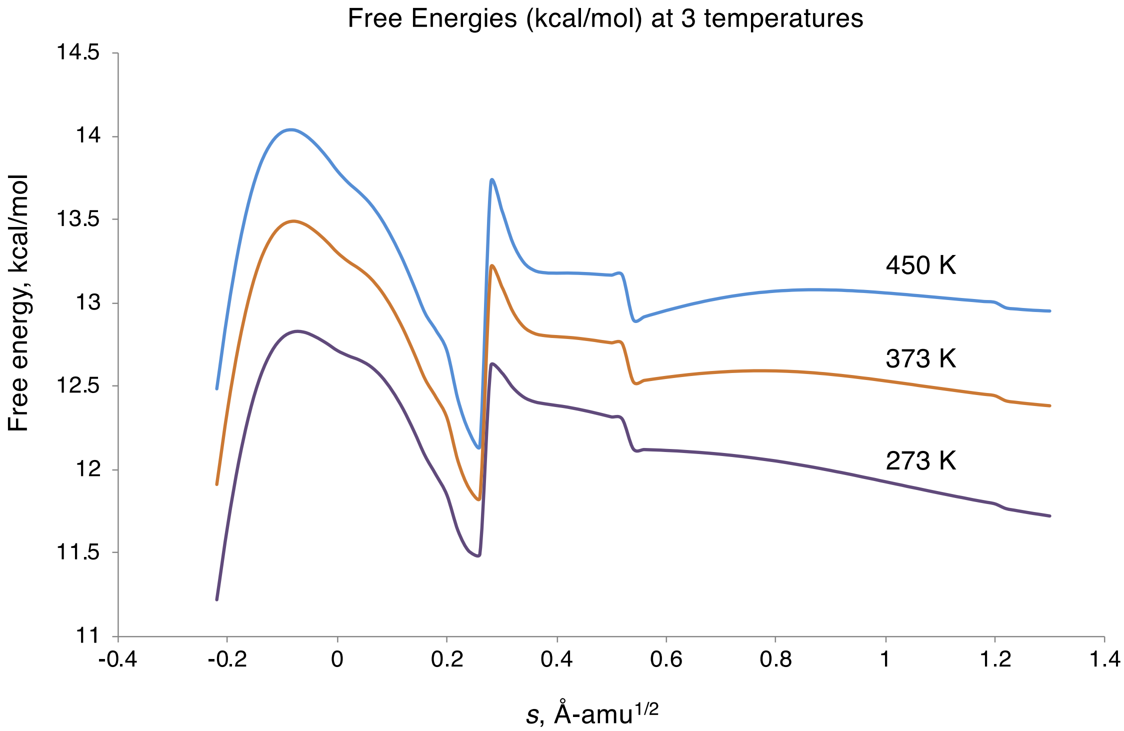

Figure S3. Free energies (kcal/mol) for tautomerization of GC to G*C* at 3 temperatures: 273, 373, and 450K, using ωB97XD/6-311+G(d,p).

For the tautomerization of GC to G**C**, notice that there are three sets of peaks at three temperatures. The peak that is close to the saddle point stays at the saddle point and the position of the peak corresponding to the quantum barrier is also retained at the same s value at the three temperatures: 273 K, 373 K, and 450 K.

Scalar curvature analysis for the tautomerization of GC to G*C* and *dd*GC to *dd*G*C* using ωB97XD/6-311+G(d,p)

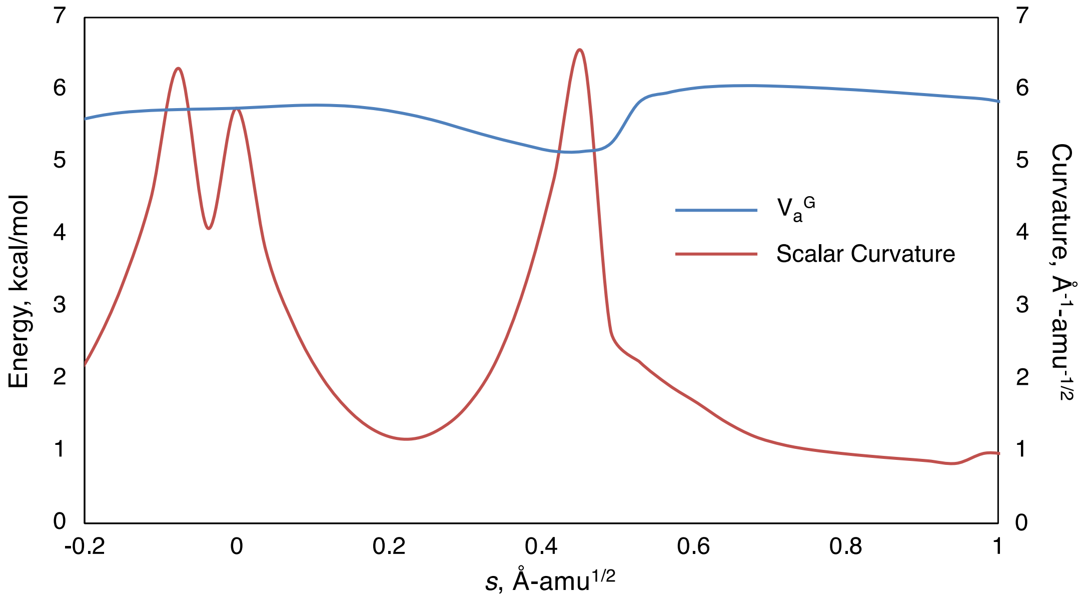

Figure S4. Scalar curvature (red curve) and V_a^G (blue curve) for the GC → G*C* normalized at s = 0 for comparison. Curvature units are Å^{-1}-amu$^{-1/2}$.

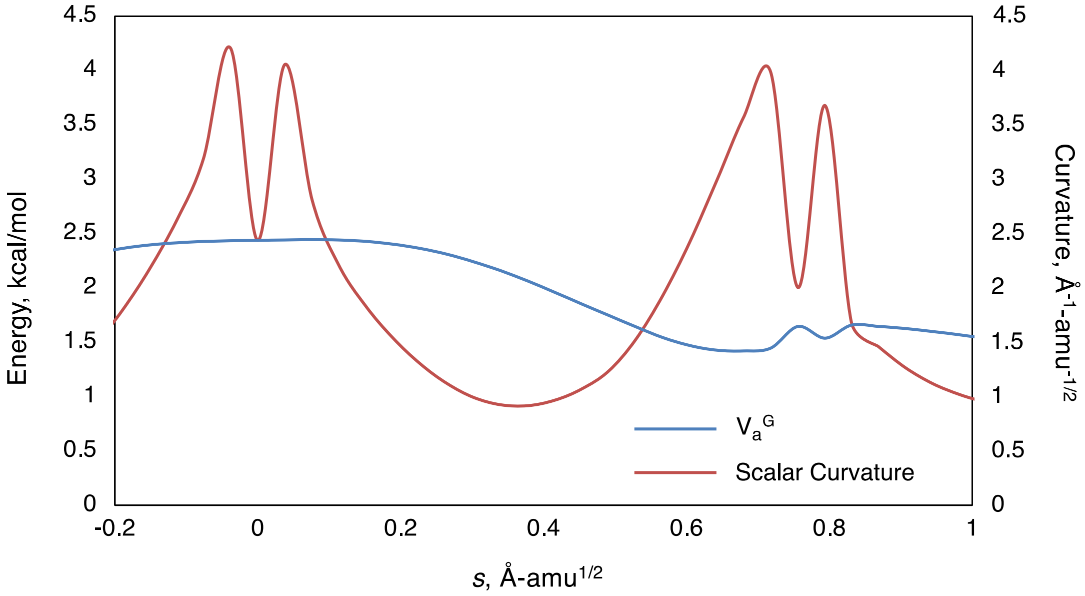

Figure S5. Scalar curvature (red curve) and V_a^G (blue curve) for the *dd*GC → *dd*G*C* normalized at s = 0 for comparison. Curvature units are Å^{-1}-amu$^{-1/2}$.

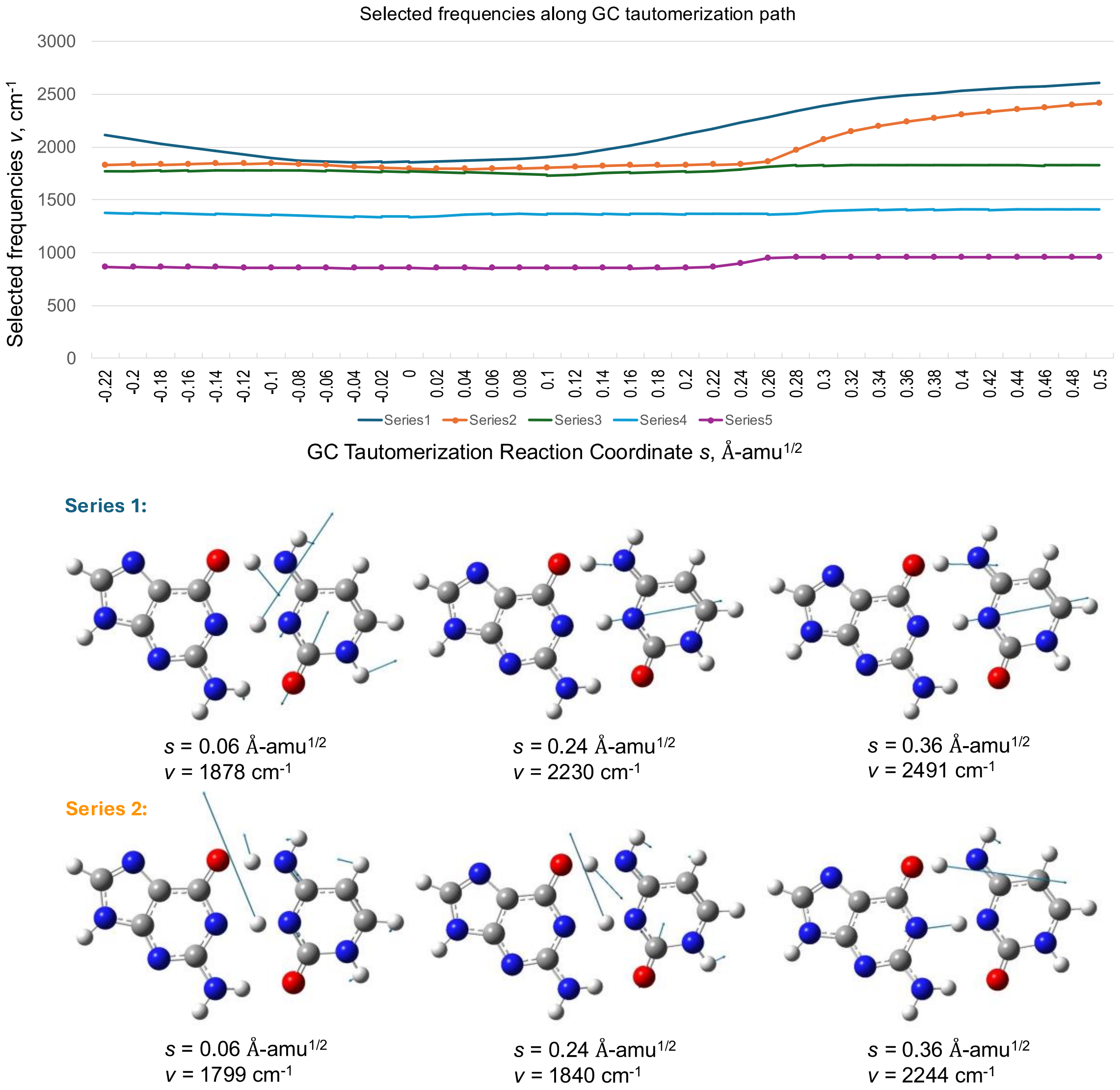

Figure S6. Selected frequencies [cm^{-1}] along GC tautomerization path [Å-$amu^{1/2}$] with eigenvectors (blue atoms = N, red = O, gray = C, light gray = H).

Starting at the saddle point, $s = 0$, the minimum energy path (MEP) is propagated in the forward direction toward the product and again, starting at $s = 0$, in the reverse direction toward the reactant. Analytical second derivatives of energy with respect to arc length s along the path are computed at regular intervals along the MEP. This gives the vibrational zero-point energy (ZPE) at each step and improves the accuracy of the path. Note that only the transverse modes (orthogonal to the path direction) contribute to ZPE. As the MEP leaves the TS region and approaches the product, the path tangent changes direction (curves) toward the product. MEP curvature and ZPE are both defined in terms of second derivatives.[43] In fact, reference 43 contains a simple math relationship between curvature and force constant matrix.

Accordingly, the presence of substantial ZPE in a region of the MEP with large curvature is reasonable.

POLYRATE prints out all frequencies at each point. We analyzed the transverse vibrational modes along the GC tautomerization pathway and identified two modes that change significantly along the reaction coordinate, referred to as Series 1 (blue) and Series 2 (orange) in Figure S6. These frequencies have the greatest effect on the ZPE, as shown in Figure S6. In Series 1 (blue), we selected three points along the reaction coordinate and examined their corresponding eigenvectors (shown with arrows in Figure S6). At s = 0.06 Å-amu$^{1/2}$, which lies in the region of the first barrier, the frequency ν = 1878 cm^{-1} corresponds to strong N–H and weak O–H wagging modes. At s = 0.24 Å-amu$^{1/2}$, just before the quantum barrier, the frequency ν = 2230 cm^{-1} evolves to high-frequency N–H and lower-frequency O–H stretching modes. At s = 0.36 Å-amu$^{1/2}$, in the region of the quantum barrier, the same frequency ν = 2491 cm^{-1} corresponds again to the combination of high-frequency N–H and significantly higher-frequency O–H stretching modes.

In Series 2 (orange), we similarly examined three points along the reaction coordinate. At s = 0.06 Å-amu$^{1/2}$ and s = 0.24 Å-amu$^{1/2}$, the frequencies ν = 1799 cm^{-1} and ν = 1840 cm^{-1}, respectively, correspond to strong N–H and weaker O–H wagging modes. At s = 0.36 Å-amu$^{1/2}$, within the quantum barrier region, the frequency ν = 2244 cm^{-1} corresponds to high-frequency N–H and much higher-frequency O–H stretching modes. Originally corresponding to the reaction coordinate, this mode has evolved into a transverse vibrational mode, which contributes to ZPE, which constitutes the quantum barrier.

Sample input files for POLYRATE calculations:
File GC.dat

```
*General

  TITLE
  GC-Tunneling
  END

  ATOMS
    1    C
    2    C
    3    C
    4    C
    5    C
    6    H
    7    H
    8    O
    9    N
   10    N
   11    N
   12    N
   13    H
   14    H
   15    N
   16    C
   17    C
   18    C
   19    C
   20    H
   21    H
   22    N
   23    H
   24    H
   25    N
   26    N
   27    O
   28    H
   29    H
  END

  NOSUPERMOL
  writefu31

*SECOND

  HESSCAL    HHOOK

*OPTIMIZATION

  OPTTS      OHOOK
  OPTMIN     OHOOK

*REACT1

   status  2
```

```
  GEOM
    1   -3.25415000  -0.61803800  -0.03243100
    2   -2.81137800   0.69758800  -0.05549100
    3   -1.40319200   0.92744000  -0.02917800
    4   -1.23984100  -1.53214500   0.03644800
    5   -4.92564000   0.81596700  -0.10663100
    6    0.33499000  -0.18041100   0.03807500
    7   -5.94938900   1.15854400  -0.13859700
    8   -0.79629500   1.99630700  -0.04215400
    9   -0.69165600  -0.27717900   0.01727500
   10   -2.53690100  -1.75624100   0.01254300
   11   -3.87457000   1.57687500  -0.10184400
   12   -4.61553200  -0.52877400  -0.06572200
   13    0.63416200  -2.43966600   0.10083400
   14   -0.77376700  -3.48606400   0.09578500
   15   -0.37777300  -2.56377500   0.08226000
   16    2.76510300   1.31056200   0.05452900
   17    4.19640800   1.48183100   0.08144200
   18    4.94425700   0.36142400   0.12556800
   19    2.95936100  -1.01846000   0.11629700
   20    4.65047300   2.46192400   0.06706200
   21    6.02662700   0.37368600   0.14890700
   22    2.20364200   0.10404900   0.07230700
   23    2.34898000   3.29697800  -0.00442100
   24    0.93626900   2.24817200  -0.00915500
   25    1.96220700   2.37022300   0.01088100
   26    4.35010400  -0.85823000   0.14273200
   27    2.50079000  -2.15471800   0.13412100
   28    4.89038300  -1.70835200   0.17586400
   29   -5.25237500  -1.30750200  -0.06048200
 END

  SPECIES   NONLINRP

*PROD1

   status  2

  GEOM
    1   -3.25071400  -0.60967800  -0.02774300
    2   -2.79205300   0.70273700  -0.08336800
    3   -1.39741000   0.85620200  -0.02437300
    4   -1.21564300  -1.45868100   0.12949400
    5   -4.90064900   0.84094400  -0.19716000
    6    1.20510200  -0.10621000   0.07127600
    7   -5.91937700   1.19332500  -0.26912800
    8   -0.86639900   2.04990400  -0.07003700
    9   -0.62843200  -0.23216400   0.07753800
   10   -2.52224400  -1.71802400   0.07587300
   11   -3.84556400   1.59470900  -0.18907200
   12   -4.61209900  -0.50717700  -0.10270200
   13    0.61524100  -2.43287000   0.15644600
   14   -0.80600800  -3.43104700   0.19559900
   15   -0.38725700  -2.52143300   0.27086400
   16    2.69835900   1.33710300   0.09934100
   17    4.14476500   1.48883000   0.10996100
```

```
  18    4.91878500   0.39444100   0.07632900
  19    3.01069400  -1.09628100   0.02912600
  20    4.57742600   2.47797800   0.14155300
  21    6.00018000   0.44097600   0.07965800
  22    2.24075800   0.02860100   0.06513800
  23    2.23896300   3.19314200   0.14222900
  24    0.13098900   2.04907900   0.00882600
  25    1.81956100   2.27093000   0.11643200
  26    4.37664000  -0.86416700   0.03357700
  27    2.56105300  -2.22612400  -0.00438300
  28    4.95616500  -1.68720500   0.01037500
  29   -5.26196400  -1.27459100  -0.08658700
 END

 SPECIES   NONLINRP

*START

 status  2

 GEOM
   1   -3.14222700  -0.39749200   0.00686000
   2   -2.66172000   0.88831400  -0.18871100
   3   -1.25348800   1.05505700  -0.16837900
   4   -1.12841400  -1.28394200   0.20589100
   5   -4.77214800   1.05826800  -0.26604500
   6    0.94660800   0.05685400  -0.02686400
   7   -5.78599900   1.42191400  -0.34975100
   8   -0.68416900   2.17558600  -0.31264400
   9   -0.51715400  -0.08201900   0.02377700
  10   -2.43519600  -1.51433700   0.20627900
  11   -3.70165400   1.78573400  -0.35558500
  12   -4.50320400  -0.27877200  -0.04570200
  13    0.64952700  -2.31671300   0.13993200
  14   -0.77680900  -3.24488800   0.39914500
  15   -0.31583400  -2.35216800   0.44572200
  16    2.57637200   1.49280900   0.13403800
  17    4.00406900   1.68349400   0.23225400
  18    4.79970600   0.60714800   0.10986200
  19    2.90223600  -0.87032500  -0.16871900
  20    4.41211200   2.67122400   0.38671100
  21    5.87940600   0.66486800   0.16135600
  22    2.09993900   0.22926500  -0.04227200
  23    2.09381200   3.39594000   0.32870600
  24    0.57684400   2.30786900  -0.02606700
  25    1.71810800   2.46897800   0.20109700
  26    4.27147200  -0.63061800  -0.09462800
  27    2.48919800  -2.00081900  -0.33464800
  28    4.85616600  -1.44633400  -0.18571300
  29   -5.16361400  -1.02953000   0.06178300
 END

 SPECIES   NONLINTS

 *PATH

    dlx3        0.005
```

```
    SCALEMASS     1.0
    RODS
#   norods
    SSTEP         0.002
    INH           10
    NSTEPS        99999
    CURV          dhess
    RPM           pagem
    FIRSTSTEP     cubic
    SIGN          reactant

    COORD         curv3

    SRANGE
       slp    3.10
       slm   -2.10
    END

   prsaverp

   specstop
    CURVE vag
    POINT savegrid
    percentdown  95.
   end

#  sfirst
#   nfstep  20
#   fsize  0.001
#  end

  INTDEF
  2-1
  3-2       3-2-1
  4-1       4-1-2       4-1-2-3
  5-2       5-2-1       5-2-1-4
  6-3       6-3-2       6-3-2-1
  7-5       7-5-2       7-5-2-1
  8-3       8-3-2       8-3-2-1
  9-4       9-4-1       9-4-1-2
 10-4      10-4-1      10-4-1-2
 11-5      11-5-2      11-5-2-1
 12-1      12-1-10     12-1-10-4
 13-4      13-4-1      13-4-1-10
 14-4      14-4-1      14-4-1-10
 15-4      15-4-1      15-4-1-10
 16-8      16-8-3      16-8-3-2
 17-16     17-16-8     17-16-8-3
 18-17     18-17-16    18-17-16-8
 19-16     19-16-8     19-16-8-3
 20-17     20-17-16    20-17-16-8
 21-18     21-18-17    21-18-17-16
 22-16     22-16-8     22-16-8-3
 23-16     23-16-8     23-16-8-3
 24-8      24-8-3      24-8-3-2
 25-16     25-16-8     25-16-8-3
 26-18     26-18-17    26-18-17-16
```

```
 27-19    27-19-16   27-19-16-8
 28-26    28-26-18   28-26-18-17
 29-12    29-12-1    29-12-1-10
  END

 *TUNNEL

    ZCT
    ALLEXCIT
    SCT
   SCTOPT
     lagrange  4
   END

   LCTOPT
     lcgmethod 4
     ilct2d
     nolctrst
     lctstr
     ngtheta   120
     ngamp      20
   END

   LCTGRID
     state 0
      9 11
   END

   QUAD
     nqe        10
     nqth       40
     NSEGBOLTZ   1
     NSEGTHETA   1
   END

*RATE
  TST
  CVT
  # CUS 2
  # PRDELG ON
  # PRGIGT ON
  # ICVT   ON

 Temp
  200.00
  225.00
  250.00
  273.15
  293.00
  298.15
  308.15
  313.00
  323.00
  333.00
  343.00
  353.00
  363.15
```

```
  373.00
  383.15
  400.00
  413.15
  438.15
  450.00
   END
```

File GC.70

```
*GRGENERAL

  GRRESTART

*GRSTART
  charge 0
  multiplicity 1

*GRREACT1
  charge 0
  multiplicity 1

*GRPROD1
  charge 0
  multiplicity 1

*GRCOMMON

  GRENER
    %mem=96GB
    %nprocshared=32
    # wb97xd/6-311+G(d,p) FCHK UNITS=AU nosymm int=SuperFineGrid
  END

  GRFIRST
    %mem=96GB
    %nprocshared=32
    # wb97xd/6-311+G(d,p) FORCE FCHK UNITS=AU nosymm int=SuperFineGrid
  END

  GRSEC
    %mem=96GB
    %nprocshared=32
    # wb97xd/6-311+G(d,p) FREQ=NORAMAN FCHK UNITS=AU nosymm
int=SuperFineGrid
  END

*GRPATH

  GRENER
    %mem=96GB
    %nprocshared=32
    # wb97xd/6-311+G(d,p) FCHK UNITS=AU nosymm int=SuperFineGrid
  END

  GRFIRST
```

```
    %mem=96GB
    %nprocshared=32
    # wb97xd/6-311+G(d,p) FORCE FCHK UNITS=AU nosymm int=SuperFineGrid
  END

  GRSEC
    %mem=96GB
    %nprocshared=32
    # wb97xd/6-311+G(d,p) FREQ=NORAMAN FCHK UNITS=AU nosymm
int=SuperFineGrid
  END
```
